# Differentiation of Cells Isolated from Afterbirth Tissues into Hepatocyte-Like Cells and Their Potential Clinical Application in Liver Regeneration

**DOI:** 10.1007/s12015-020-10045-2

**Published:** 2020-09-25

**Authors:** Marcin Michalik, Aleksandra Gładyś, Piotr Czekaj

**Affiliations:** Department of Cytophysiology, Faculty of Medical Sciences in Katowice, Medical University of Silesia in Katowice, Katowice, Poland

**Keywords:** Afterbirth cells, Differentiation, Hepatocytes, Liver failure, Cell transplantation

## Abstract

Toxic, viral and surgical injuries can pose medical indications for liver transplantation. The number of patients waiting for a liver transplant still increases, but the number of organ donors is insufficient. Hepatocyte transplantation was suggested as a promising alternative to liver transplantation, however, this method has some significant limitations. Currently, afterbirth tissues seem to be an interesting source of cells for the regenerative medicine, because of their unique biological and immunological properties. It has been proven in experimental animal models, that the native stem cells, and to a greater extent, hepatocyte-like cells derived from them and transplanted, can accelerate regenerative processes and restore organ functioning. The effective protocol for obtaining functional mature hepatocytes *in vitro* is still not defined, but some studies resulted in obtaining functionally active hepatocyte-like cells. In this review, we focused on human stem cells isolated from placenta and umbilical cord, as potent precursors of hepatocyte-like cells for regenerative medicine. We summarized the results of preclinical and clinical studies dealing with the introduction of epithelial and mesenchymal stem cells of the afterbirth origin to the liver failure therapy. It was concluded that the use of native afterbirth epithelial and mesenchymal cells in the treatment of liver failure could support liver function and regeneration. This effect would be enhanced by the use of hepatocyte-like cells obtained from placental and/or umbilical stem cells.

**Figure:**
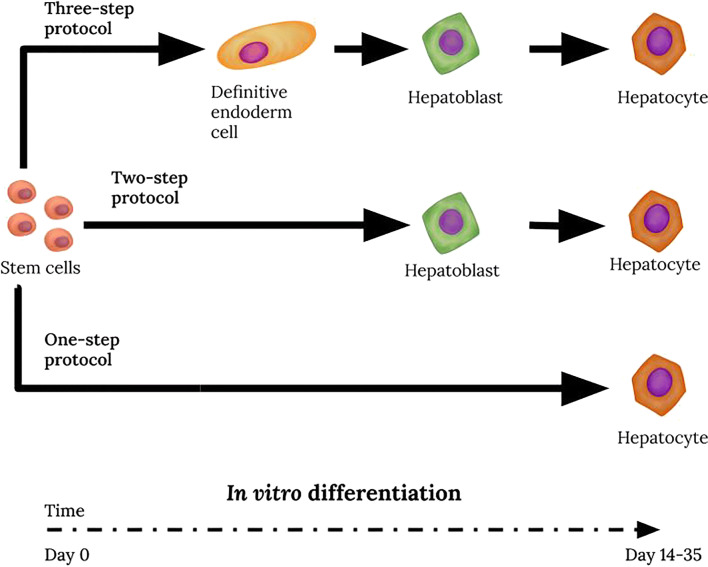
Graphical abstract

Graphical abstract

## Introduction

The number of patients waiting for a liver transplant increases every year. This is caused, among other reasons, by clinically justified transplant indications and insufficient number of organ donors. As a consequence, the waiting time for the transplantation is getting longer, leading to a shorter transplant functioning time, and lower patient survival rate [1]. Another unfavorable prognostic phenomenon is an increase in donors' age, resulting in transplantation of organs with a shorter expected functioning time [2]. A partial solution for the growing demand for liver transplantations was the introduction of a ‘split’ procedure, in which two parts of the graft are separated: left lateral segment graft and right extended liver lobe graft. Due to this procedure, it is possible to partially meet the demand and also to obtain the transplants of reduced volume for pediatric recipients [3]. Another promising alternative to the whole organ transplantation procedure is the hepatocyte transplantation. It is currently being evaluated in some clinical trials, where hepatocytes are transplanted to patients with hepatic failure, thus restoring their population [4]. However, the use of this method is very limited. Among the limitations are: difficulties with obtaining high-quality hepatocytes, insufficient colonization of the liver by transplanted cells (in particular in case of cirrhosis), and the lack of reliable data describing a long-term effect. Currently, there are only two clear indications for this procedure: inborn errors of metabolism such as Crigler-Najjar syndrome, and acute liver damage [5].

The growing demand for liver transplantation, caused by its failure and/or the need to maintain its function until the transplantation procedure, is a strong stimulus to look for alternative therapies, such as the stem cell therapy. Afterbirth tissues, i.e. placenta and umbilical cord are abundant sources of cells, that meet the biological criteria of good transplantable material, and can be isolated in a manner that does not raise moral and ethical controversies. Some of afterbirth cell populations are successfully used in clinical trials, including the treatment of type 1 diabetes, systemic lupus erythematosus and ischemic stroke [6–11]. Both *in vivo* and *in vitro* studies have shown that cells derived from these sources, e.g. amniotic epithelial cells, have potential for proliferation and differentiation, and show some efficacy in the treatment of liver failure. The use of amniotic cells in therapy of hepatic failure in animal models resulted in organ functioning improvement. Positive effect of human amnion cells (hAC) was observed in case of acquired failure caused by chemical damage and also in congenital failure caused by inborn errors of metabolism [12]. The expression profile of surface antigens enables the use of these cells in treatment of liver failure in humans without the need for immunosuppression, since they are characterized by the lack of expression of class II histocompatibility antigens. They express the CD47 antigen responsible for "don't eat me signal", and also complement antigen complex CD44/CD59 [13]. It seems that the positive effect is greater, when the used cells are close to the mature hepatocytes [14]. Therefore, the preliminary *ex vivo* differentiation of stem cells may be crucial for the effectiveness of the therapy of damaged organ applied resulting in its *in vivo* regeneration.

## Liver Development and Regeneration

The mature liver has considerable capacity for spontaneous regeneration. Several cell populations participate in this process. These are mainly hepatocytes, but also multiple multipotent and bipotent stem cell populations expressing endodermal or mesenchymal markers [15–20].

In the process of liver regeneration, the sequence of cellular changes refers to changes occurring during its embryonic development. Models of liver development are comparable among mammal species, including mice, rats, and humans [21]. In mice, hepatoblast differentiation to hepatocytes occurs between the 14th and 16th day of embryo development. In humans, this process begins around 56-58 days of pregnancy and ends around day 210 [22].

In the developing liver, there is a continuous cross-talk between endodermal, mesendodermal, and mesenchymal cells, as a result of direct action as well as paracrine signaling. These populations also stimulate hematopoietic cells inhabiting the fetal liver [17]. Liver stem cells, hepatic stellate (Ito) cells, endothelial cells, and presumably mesenchymal cells secrete growth factors stimulating hepatoblasts to proliferate and differentiate into hepatocytes or cholangiocytes [23]. At early stages of embryonic development, hepatogenesis depends on such factors, as FGF (fibroblast growth factor), BMP (bone morphogenetic protein), WNT [24], HGF (hepatocyte growth factor), MAPK9 (mitogen-activated protein kinase 9) and TGFβ (transforming growth factor β) [25]. At later stages of the prenatal period, liver development depends more on HGF-related signaling pathways, but also on insulin, cAMP (cyclic adenosine monophosphate), TGFα (transforming growth factor α), PI3K (phosphatidylinositol-3 kinase), mTOR (mechanistic target of rapamycin) and MAPK/ERK kinases [26].

Human hepatic stem cells (hHpSC) seem to be the primary population of cells with the greatest potential of proliferation and differentiation in the hepatic direction. hHpSC have been characterized as multipotent precursors to fetal hepatic progenitor cells - hepatoblasts, located in ductal plates in fetal and neonatal livers, and also in the canals of Hering in adult livers of all donor ages [27].

A population of cells with a similar or perhaps identical phenotype to the hHpSCs are biliary tree stem/progenitor cells (BTSC). BTSC are multipotent stem cells located in peribiliary glands of large intrahepatic and extrahepatic bile ducts and can be differentiated into hepatic and pancreatic lineages [28, 29]. BTSC populations are characterized by the expression of classic endodermal transcription factors: FOXA2, HNF6 (hepatic nuclear factor 6), PROX1, SLL4, SOX9, SOX17, and markers specific for endodermal progenitors: EpCAM (epithelial cell adhesion molecule), NCAM (neural cell adhesion molecule), CD133, and CXCR4. They are negative or express low levels of hepatic markers: AFP (alpha-fetoprotein), ALB (albumin), GGT (gamma-glutamyl transferase) and endocrine pancreas (insulin, glucagon), as well as mesenchymal, endothelial, and hematopoietic markers [29]. BTSC are available from all age donors and it was suggested that they may be involved in the regeneration of the liver, pancreas, and bile ducts [28].

Human hHpSC and hepatoblasts overlap in their phenotypic markers (Table 1) with prominent participation of EpCAM. EpCAM (CD326) is a multifunctional transmembrane protein that is mainly involved in intercellular adhesion. In neoplastic cells and cancer stem cells, it participates in mediating the signal for proliferation and maintaining the stem-like character of cells [30]. However, during the differentiation of hHpSC into hepatoblasts, the expression of EpCAM on their surface decreases. At the same time, on the surface of hepatoblasts the expression of NCAM (CD56) completely disappears, and it is replaced by ICAM-1 (intercellular adhesion molecule 1, CD54). Moreover, the expression of AFP, CD133, and some other markers, increases [24, 27, 31, 32]. Along with the progress of stem cell differentiation into hepatoblast, the enhanced expression of AFP, albumin, and fetal P450 isoenzymes, eg. CYP3A7, was noted in mice [33]. On the other hand, hHpSC and hepatoblasts do not express markers specific for adult hepatic progenitor cells (HPC), also known in rodents as oval cells, eg. CD34 and CD117 [27, 34].

**Table 1:** Human hepatic stem cells (hHpSC) and hepatoblasts

Positive markers	Negative markers	Ref.
Human hepatic stem cells		
**EpCAM, NCAM**, CD44H, CD133, CK8/18/19, claudin-3, CXCR4, FOX2, SOX9, SOX17, telomerase, ALB (weak or negligible)	**ICAM1, AFP**, CD45, CYP450, desmin, VEGFr,	[27, 35]
Hepatoblasts		
**EpCAM, ICAM-1, AFP, ALB,** CD29, CD44H, CD133, CK8, CK19, CYP3A7, DLK1	**NCAM,** α-SMA, CD14, **CD34**, CD38, **CD45**, CD90, CD146, CYP3A4, desmin	[17, 27, 35]
**EpCAM, AFP, ALB,** DLK1, GPC3	**NCAM, CD45**, CD235a, CK7	[36]
**EpCAM**, CD26, CD49f, CD324, CK18, CK19	**CD34, CD45**, CD133	[37]
**EpCAM, ICAM-1, AFP, ALB**, CK8, CK18, CK19, CYP3A7, IHH, PTC1, SHH, SOX-9, SOX-17	**CD34, CD45**	[31]
**EpCAM, ICAM-1, AFP, ALB**, CK8, CK18, CK19	**NCAM,** CD31, **CD34**, CD38, **CD45**, CD90, CD105, CD146, claudin-3, CYP3A4, desmin, VEGFr	[38]

hHpSC are multipotent and self-renewing precursors for bipotent prenatal hepatoblasts giving rise to mature hepatocytes and cholangiocytes, as well as to other endodermal cell types. hHpSC, hepatoblasts, and most cholangiocytes express EpCAM, whereas mature hepatocytes are EpCAM^-^. Both hHpSC and hepatoblasts do not express hematopoietic, endothelial or mesenchymal markers. In general, they are different from each other in that hHpSC are NCAM^+^/ICAM1^-^/AFP^-^, while hepatoblasts are NCAM^-^/ICAM1^+^/AFP^+^

Abbreviations: *α-SMA* α-smooth muscle actin, *IHH* Indian hedgehog, *SHH* Sonic hedgehog.

The roles of the various markers associated with liver stem and progenitor potential for differentiation was confirmed in the iPSC-based model of liver development. Elevated expression of EpCAM and HNF4-α (as well as GATA4, claudin, and NCAM) was observed in early hepatic differentiation, at the stage of hepatic stem cell right after definitive endoderm, while increased expression of AFP and CD133, as well as ICAM1, CK19 (cytokeratin 19), and SOX9 was specific for a later stage of hepatoblast-like hepatic progenitors [24].

Hepatoblasts are defined as bipotent and give rise to liver precursors for hepatocyte or cholangiocyte cell lines. Process of hepatoblast maturation is associated with changes in the expression profile of many transcription factors [25]. Mature hepatocytes are entirely negative for progenitor markers such as EpCAM, AFP, and Dlk1 (delta-like 1 homolog). In contrast, during differentiation the expression of proteins specific for mature liver, such as albumin, alpha-1 antitrypsin (A1AT), cytokeratin 18, cytochrome P450, transferrin, and tyrosine aminotransferase (TAT), increases. Moreover, these matured cells acquire the ability to synthesize and store glycogen [24]. A similar phenomenon of the loss of progenitor cell markers occurs during hepatoblasts differentiation into cholangiocytes. As a result, mature bile duct epithelial cells become positive for markers such as CK7, CK19, osteopontin, GGT and CFTR [39, 40].

Attempts to use human liver fetal cells in clinical trials have shown their safety and long-term effect in the treatment of liver cirrhosis of various etiologies, resulting in the improvement of organ function expressed by a decrease in MELD score [41]. However, the cells used in this clinical trial did not represent a homogeneous hepatoblast population. Patients received an isolated mixture of cells consisting of hepatocytic, hematopoietic and mesenchymal cell lines, expressing such markers as vimentin, CD90, and CD29. Therefore, it is not possible to give an unambiguous answer whether human hepatoblasts support adult liver regeneration in humans, or it may be the consequence of an action of other liver stem/progenitor populations. Moreover, it should be taken into consideration that the main limitation in the use of fetal liver stem cells in the treatment of liver failure on a large scale is the ethical controversy related to obtaining these cells from human fetuses.

The capability of the adult liver to regenerate after partial hepatectomy reflects the proliferative activity of the existing hepatocytes [18]. Mature hepatocytes are in the G0 phase of the cell cycle [42, 43]. Valizadeh et al. distinguish three phases in liver regeneration following a mechanical injury of the liver. Initially, it is the phase of TNFα (tumor necrosis factor-alpha) and IL-6 (interleukin 6) secretion by Kupffer cells and sinusoidal endothelial cells. As a result of their interaction, hepatocytes pass from phase G0 to G1, and then from G1 to S. Finally, under the influence of the initiating growth factors: HGF and TGFα, hepatocytes go into phase M, which results in their proliferation. In the regeneration phase, the activated liver cells pass from the G1 to M phase [42]. The regeneration phase is maintained and stimulated by direct and indirect growth factors [44]. The most important direct factors promoting this process are ligands for EGFR (epidermal growth factor receptor), namely HGF, EGF, TGFα and HB-EGF; FGF (fibroblast growth factor) and its receptors, as well as Ras-MAPK and PI3K/ACT pathways [42]. Mentioned above, direct factors promote mitosis of hepatocytes *in vitro* and *in vivo*. Among the auxiliary factors involved in liver regeneration and differentiation of hepatocytes are: bile acids, estrogens, IGF-1 (insulin-like growth factor 1), insulin, TNFα and TNFR1 (tumor necrosis factor receptor 1), IL-6, STAT3, norepinephrine, FXR receptor, TFR5 receptor, VEGF (vascular endothelial growth factor), as well as Wnt/β-catenin and Hedgehog signaling pathways [43]. Auxiliary factors do not stimulate the divisions of mature hepatocytes, but they intensify and accelerate the action of direct factors [45].

After partial hepatectomy, the liver is able to restore a significant part of its original volume (in rat liver, up to 70% of its mass may be restored) as a result of the proliferation of hepatocytes, bile duct epithelium, Kupffer cells, endothelial cells, Ito cells and fibroblasts [46, 47]. This proliferation, however, occurs not due to mechanical damage, but it is a reaction to a decrease in organ size [46]. It is known that after partial hepatectomy, human liver regenerates within 3-12 months, and the rate of regeneration is proportional to the volume of the resected fragment [48]. In the study of healthy living donors’ livers, their liver volume after right-sided hemihepatectomy increased by 94% after seven days (liver volume after surgery was 35% of the original volume), while in patients after left lateral sectionectomy the volume increased by 22% seven days after surgery (with a postoperative volume of 81% of the original one) [49]. The mechanisms for maintaining a constant liver mass have been termed "hepatostat" [46, 50, 51]. Despite restoring normal cell numbers and organ weight, the amount of liver lobules is not increased [51]. Stoppage of regenerative processes occurs mainly through the Yap protein associated with the Hippo pathway, and pathways associated with the integrin α3/β1 [43].

It is generally accepted that liver regeneration after partial hepatectomy is mediated by hepatocytes, however in the chronic liver diseases of various etiologies the regenerative potential of hepatocytes is limited, and multipotent or bipotent resident stem/progenitor cells are activated.

Multipotent hepatic stem cells can be isolated from both healthy and diseased adult human livers [52]. They are characterized by the expression of mesenchymal markers, as well as some hepatocytic, cholangiocytic and neuronal markers, integrins and adhesion molecules (Table 2). There are only few studies confirming expression of pluripotent markers in these cells. In turn, they do not express hematopoietic and endothelial markers [53, 54].

**Table 2:** Phenotypic characteristics of adult-derived human liver multipotent stem cells expressing typical mesenchymal markers

Original cell name	Positive markers	Negative markers	Hepatogenic differentiation	Differentiation potential	Ref.
Human liver stem-like cells (HLSC)	*Mesenchymal:* CD44, CD73, CD90, CD105 (20%), CD146 (18%), vimentin *Other:* AFP, ALB, CD29, CD31, CK8 (11-30%), CK18 (15-30%), HLA-I, HLA-G, Musashi1, NANOG, nestin, Oct-4, Pax2, Sox2, SSEA4	*Mesenchymal:* α-SMA, STRO-1 *Other*: CD14, CD31, CD34, CD45, CD117, CD133, CK19, cytochrome P450, NCAM	Rotary Cell Culture System; DMEM low glucose, 40% MCDB-201, 1x ITS, 1x linoleic acid 2-phosphate, 10^−9^ M dexamethasone, 10^−4^ ascorbic acid 2-phosphate, 10 ng/ml HGF, 10 ng/ml FGF4, 2% FBS or Rotary Cell Culture System; α-MEM/EBM 3:1, 12 mM Hepes, 2% FCS, 10 ng/ml HGF, 10 ng/ml FGF4 or rat liver acellular scaffolds recellurized with HLSC in standard medium or with 10 ng/ml EGF and 10 ng/ml FGF4, or with HLSC-derived conditioned medium	hepatogenic, osteogenic, endothelial, insulin-producing islet-like structures no adipogenic potential	[52–60]
	*Mesenchymal:* CD29, CD44, CD73, CD44, CD90, CD105, CD166 *Other*: CK8, CK18, HLA-I	CD14, CD34, CD38, CD45, CD62L, CD117, CD144, CK19, HLA-II	1st step: DMEM, 1% ITS, 10 ng/mL FGF-1, 10 ng/mL FGF-4, 20 ng/mL HGF (5 days) 2nd step: DMEM, 100 nM dexamethasone, 10 ng/mL FGF-4, 20 ng/mL HGF, 10 ng/mL oncostatin M, 0.5% DMSO or 1st step: IMDM, 2% FBS, 10 ng/mL FGF-4, 20 ng/mL HGF, and 5 μmol/L nicotinamide (7 days) 2nd step: IMDM, 2% FBS, 10 ng/mL FGF-4, 20 ng/mL oncostatin M, 1 μmol/L dexamethasone, 50 g/mL ITS Premix, μmol/L trichostatin A parallel differentiation protocol: addition of 2.5 mmol/L sodium butyrate in step 1 and 2.5 mmol/L sodium butyrate plus 20 ng/mL HGF in step 2	hepatogenic, octeogenic, chondrogenic no adipogenic potential	[61–63]
Adult-derived human liver mesenchymal-like cells (ADHLSC)	*Mesenchymal:* α-SMA, CD13, CD44, CD73, CD90, vimentin *Other:* ALB, CD29, CD49b (32%), CD49e (31%), HLA-I; *at mRNA level:* A1AT, AFP, CYP1B1, CYP3A4, G6P, GGT, GS,HNF-4α, MRP2.	*Mesenchymal:* CD105 *Other:* CD49f (19%), CD34, CD45, CD117, CD133, CK7, CK8, CK18, CK19, CYP2B6, HLA-II, Oct-4, TAT, TDO	1st step: IMDM, 20 ng/ml HGF, 10 ng/ml bFGF, nicotinamide 0.61 g/L, 1% ITS (10 days) 2nd step: IMDM, 20ng/ml oncostatin M, 1 μM dexamethasone, 1% ITS (10 days)	hepatogenic no osteogenic or adipogenic potential	[64]
Liver-derived mesenchymal stem cells (L-MSCs)	*Mesenchymal:* CD90, CD105, CD167 *Other*: CK19, HGF	ALB, CD34, CD45, CK18, c-Met, HLA-II	1st step: IMDM with 20 ng/mL EGF, 10 ng/mL FGF (2 days) 2nd step: IMDM with 20 ng/mL HGF, 10 ng/m LFGF, 0.61 g/L nicotinamide, 1% ITS (10 days) 3rd step: incubation with infectious Japanese fulminant hepatitis 1 (JFH1)–derived hepatitis C virus (HCV) particles (10 days)	hepatogenic, adipogenic, osteogenic	[65]
Liver Mesenchymal Stem Cells/Liver Stromal Cells	*Mesenchymal:* CD44 (30%), CD90 (30%) *Other****:*** CD166 (10-25%), CD227 (10-25%), c-Met (50%)	CD34, CD45, CD91, EpCAM	1st step: DMEM/F12, 2 mM GlutaMAX, 0.5% FBS, 10 ng/mL HGF, 100 ng/mL Activin A (10 days) 2nd step: DMEM/F12, 2 mM GlutaMAX, 15 mM HEPES, 2 μg/mL insulin, 10 ng/mL FGF-4, 10 ng/mL HGF, 10 ng/mL oncostatin M, 10^–7^ M dexamethasone (10 days)	hepatogenic	[15, 66, 67]

One exception is CD105 which expression was shown to be low or absent in some studies. Liver mesenchymal stem cells do not express hematopoietic and endothelial markers, and are different from bipotent hepatic progenitor cells (HPC)

Abbreviations: *GS* glutamine synthase, *ITS* insulin, transferrin and selenous acid, *MRP2* multidrug resistance protein-2, *TDO* tryptophan 2,3-dioxygenase, *HLA* human leukocyte antigen

In *in vitro* culture stem cells expressing mesenchymal markers have spindle-shaped morphology resembling that of mesenchymal stem cells (MSC) isolated from other human tissues. Their location within the liver parenchyma is not precisely determined, and an origin is not known. It was hypothesised that these stem cells are the descendants of resident MSC persisting in the liver, circulating MSC or bone marrow mesenchymal stem cells (BM-MSC), hepatic pericytes or Ito cells, or dedifferentiated hepatocytes by the process of epithelial-to-mesenchymal transition [15].

*In vitro* liver mesenchymal stem cells divide intensively reaching the confluence required for passage after about 3-5 days of culture. They are able to differentiate *in vitro* not only towards the hepatocytic lineage [52, 59, 60, 64, 65], but also osteocytic [52, 59, 65], chondrocytic [61], endothelial [52, 53] lineages, and insulin-producing islet cells of the pancreas [52, 56], but not to the adipocytic lineage [52, 59, 61]. On the other hand, one study demonstrated that liver mesenchymal stem cells are able the differentiate into adipocyte-like cells [65]. Following intrasplenic administration to mice, hepatic stem cells were localized in the liver and differentiated into hepatocytes *in vivo*, thus acquiring the ability to synthesize albumin [64].

The therapeutic potential of human liver stem cells and extracellular vesicles (EVs) derived from these cells has been reported in numerous preclinical animal trials, and subsequently also in human clinical trials. Preclinical trials with the use of human liver stem-like cells (HLSC) revealed increased survival of mice with Crigler-Najjar syndrome type 1 [60] and acute liver failure [52, 55], and alleviated the symptoms of nonalcoholic fatty liver disease in mice [57, 68]. Moreover, HLSC improved the function of acutely damaged kidneys of various etiologies [69]. Animal models showed the protective effect of HLSC-EVs in chronic kidney disease and the lower expression of genes responsible for kidney fibrosis [70]. EVs reduced liver damage in a mouse model of liver injury associated with hepatic perfusion disorders [71] and reversed kidney damage in mice with diabetic nephropathy [72]. The therapeutic potential of HLSC-EVs was demonstrated also in cancer cell research. When applied *in vivo* to mice, HLSC-EVs influenced human kidney cancer cells by reducing their activity, inducing apoptosis and inhibiting proliferation. Furthermore, by reducing the degree of tumor vascularization, the EVs increased the time needed to the occurrence of metastases [58, 73, 74].

Successful preclinical trials of the use of HLSC in cell therapy resulted in their admission to the first phase of clinical trials in a study of metabolic hyperammonemia in children. The results confirmed the safety of HLSC administration to humans. No significant negative side effects were observed, and no need for immunosuppression was demonstrated [59]. Several human clinical trials using HLSC are currently ongoing. Attempts are being made to use them in various liver diseases: congenital, as well as acquired, such as chronic liver failure or NASH (NCT03884959, NCT02946554, NCT03963921, NCT01765283) [75].

Human bipotent hepatic progenitor cells (HPC) are often referred to as oval cells due to their shape in rodents livers. HPC are quiescent in healthy liver and become precursors of hepatocytes and cholangiocytes in injured liver [76, 77]. This is a reason that many markers of HPC are only expressed in the liver after chemical or viral injury, when these facultative progenitor cells are activated. The number of HPC correlates with the severity of liver disease [78].

Oval cells/HPC are located in the wall of canals of Hering in periportal areas. Proliferation and differentiation of oval cells was observed in rat and mouse models of chronic liver injury in which hepatocyte proliferation is inhibited. HPC expand in human chronic liver diseases, mainly chronic inflammations of various etiologies and tumorigenesis [79–81]. In humans, a minimum 50% hepatocyte loss is required for significant activation of the HPC compartment [78]. Wnt pathway plays a significant role in HPC expansion, while the Notch pathway is involved in the HPC differentiation towards the cholangiocytic lineage [82].

The process of the liver regeneration dependent on oval cells/HPC is often referred to as ductal reaction. Besides its activation, ductal reaction also includes immune cell infiltration, Ito cells activation and remodeling of the extracellular matrix - ECM [83]. In a damaged liver, Kupffer cells secrete pro-inflammatory cytokines such as TNFα and IL-6 in response to complement activation or under the influence of the superantigen - LPS (lipopolysaccharide). These ligands, by connecting with their IL-6R and TNFR1 receptors on progenitor cells, stimulate their proliferation [84], and lead to activation of MAPK and PI3K/AKT signaling pathways [42] involved, among others, in hepatogenic differentiation [84, 85]. Progenitor cells proliferate and then migrate to Disse's space or even liver parenchyma [86], where they differentiate into hepatocytes or cholangiocytes [87]. Concentration of proinflammatory cytokines including INFɣ (Interferon-gamma), increases in damaged liver parenchyma. Inflammatory cytokines stimulate M1 macrophages to secrete pro-inflammatory and profibrotic cytokines. In contrast, M2 cells secrete IL-4 and IL-13 factors, that inhibit the inflammatory response and participate in extracellular matrix degradation [88].

The cellular origin of heterogenous population of HPC is still not clarified. The relationship between HPC that appear in the adult liver and fetally derived hHpSC remains unresolved. In turn, based on the comparison of HPC and fetal hepatoblasts, which both are proliferating, clonogenic, bipotential, and share the same markers such as Dlk-1, and alpha-fetoprotein, as well as are able to repopulate *in vivo*, it has been suggested that hepatoblasts are precursors to HPC. On the other hand, in some studies the cells expressing Dlk-1 and AFP have not been observed in the healthy, uninjured liver, what argues against the relationship between fetal hepatoblast and adult HPC [78]. Furthermore, HPC differ phenotypically and functionally from human liver multipotent stem cells expressing mesenchymal markers [52, 59]. These contradictory findings regarding the origin of HPC may also suggest their multiple maturational stages [38, 78].

A pattern of specific markers for HPC determined in patients with chronic liver injury or submassive hepatic necrosis was shown to be similar to those of rodent oval cells. Commonly used markers for the identification of oval cells in rodents are, among others: adult hepatocyte markers (A1AT, ALB, CK8, CK18, HNF4), hepatoblast markers (AFP, CD26, CD29, CD49f, GGT, MPK, and some other), cholangiocyte markers (A6 antigen, CD133, C-met, CK7, CK19, EpCAM, OV-1, OV-6), hematopoetic markers (CD34, c-Kit, CXCR4, Thy-1), and neuroepithelial markers as chromogranin A. Besides, on their surface, there are receptors for HGF, EGF, and TNFα, which are involved in signal transmission to proliferate and differentiate [22, 76, 77]. A distinct pattern of HPC surface markers was found between acute and chronic liver diseases [89]. Most of the molecular markers for oval cells are also expressed in cholangiocytes [76]. As a consequence, there are no specific markers to differentiate HPC from cholangiocytes and the application of the above markers seems to be limited by their lack of cell-type specificity [83].

The application of HPC to cell therapy is an attractive solution, because HPC can be isolated even from diseased livers, expanded in culture without losing its bidirectional differentiation potential, and transplanted back to the patient without the need for immunosuppression [78]. Unfortunately, the efficiency of HPC repopulation and engraftment is still relatively low compared to primary hepatocyte transplantation. Current protocols fail to differentiate HPC into fully mature hepatocytes in culture, and it is not known whether HPC can be tumorigenic in the recipient [78, 83].

It should be taken into consideration that the thesis that facultative liver stem cells are an important source of new hepatocytes and cholangiocytes in chronically injured liver is being challenged [16, 90–92]. It cannot be excluded that also the plasticity of both hepatocytes and cholangiocytes can account for tissue repair in the liver and biliary regeneration, including highly replicative hepatocyte subpopulations, hybrid hepatocytes, hepatocytes characterized by high expression of telomerase, as well as so-called small cholangiocytes. Finally, transdifferentiation of both hepatocytes and cholangiocytes into the other compartment when one compartment is significantly damaged was observed [83, 93]. It also was suggested that hepatocytes can dedifferentiate into HPC in response to liver injury [78].

As in many other cells with a stemness potential, the activation of hepatoprogenitors carries the risk of neoplastic transformation, that gives rise to an immortal cell line [77]. However, the regenerating liver has inhibitory mechanisms necessary to avoid excessive proliferation resulting in neoplasia. Inhibitory factors include TGFβ and - belonging to the same family: BMP and Activin A. The inhibitory effect of TGFβ on hepatocyte proliferation is balanced by the profibrotic effects of Activin A and Activin B, as well as BMPs. At high concentrations, profibrotic factors from the TGFβ family affect Ito cells and stimulate them to transform into myofibroblasts, which results in increased liver fibrosis, cirrhosis and hepatocyte death [94]. Activin A often appears as a supplement in hepatocyte differentiating media, despite its proven proapoptotic properties against hepatocytes [95].

## Differentiation of Afterbirth Cells Towards Hepatocytes

To date, a number of attempts have been made to differentiate stem cells into hepatocytes, with the intention of using them in organ regeneration. Some satisfactory results have been obtained in *in vitro* differentiation of cells such as human embryonic stem cells (hESC) [96, 97], human dental pulp stem cells (hDPSC) [98, 99] and bone marrow mesenchymal stem cells [100–102], as well as placental and umbilical cord stem cells (Tables 3, 4 and 5).

**Table 3:** Protocols for *in vitro* hAEC differentiation towards hepatocytes, ordered by culture time. Observed changes in gene and protein expression are included

Culture time & surface	Differentiating medium	Gene up-regulation	Gene down-regulation	Protein expression	Cell activity	Ref.
14 days Matrigel	IMDM, 10% Fetal bovine serum (FBS), 100 mM non-essential amino acids (NEAA), 2 mM L-glutamine, 10 ng/ml EGF, 10 ng/ml bFGF, 20 ng/ml HGF, 1 μM dexamethasone (dex), 20 ng/ml oncostatin M (OSM), 55 μM β-mercaptoethanol, 1% ITS Premix	A1AT, AFP, ALB, CK19, CYP: 1A2, 2B6, 3A4, 7A1	NANOG, OCT-4, SSEA4	ALB	Indocyanine green uptake, glycogen storage, albumin and urea secretion	[113]
18 days plastic	DMEM/B27, 10ng/mL FGF2, 20ng/ml HGF, 100ng/ml Activin A, 20ng/ml OSM, 100μm/L Sodium Taurocholate Hydrate, 20ng/ml BMP4	AFP, CYP7A1, FOXA1, TAT	OCT-4	A1AT, ALB, HNF-4α	Indocyanine green and LDL uptake, CYP450 inducible activity	[14]
20 days Collagen type-I	DMEM/IDMEM, 10% FBS, 20ng/ml EGF, 10^-7^M dex, 100ng/ml Activin-A, 0,5% m Hu-alb (Human albumin)	A1AT, ALB, CAR, C/EBPa, C/EBPb, CK8, CK18, CK19, c-MET, CYP: 1A2, 2B6, 2C8, 2C9, 2C19, 2D6, 3A4, 3A7, 7A1, HNF-1α, HNF-4α, OATP, PPAR, PXR, RAR, RXR,	BRCP, OCT-4	Not given	Not given	[114]
~21 days L-ECM	IDMEM, 5% FBS, 10ng/ml EGF, 10ng/ml FGF2, 10ng/ml HGF, 10^−6^ M dex, 100ng/ml Activin A	ALB, CYP: 2B6, 2D6, 3A4, 3A7, UGT1A1	Not given	ALB, CYP2E1, CYP3A1	Ammonia metabolism, CYP450 inducible activity	[115]
~28 days plastic	IMDM, 10% FBS, 1 mM NEAA, 4 mM L-glutamine, 10 ng/ml EGF, 0.1 μM dex, 1 mM pyruvate	A1AT, ALB, AFP, CCND1, CYP7A1	NANOG, OCT-4, P21, P53, SOX-2	CYP3A4, CYP7A1,	Increased ERK 1/2 phosphorylation	[85]
~35 days Collagen type-I	DMEM/F12, 10% FBS, 10ng/ml EGF, FGF-4, HGF, 0,1μM dex, 10% HepG2 cell-conditioned medium, 0,1μM insulin	ABCA2, ABCB11, ASS1, CYP3A4, CYP7A1	EPHX1, SLC27A	ALB, HNF-4α	LDL and glycogen presence, Indocyanine green uptake, urea synthesis	[116]

**Table 4:** Protocols for *in vitro* differentiation of placental MSC towards hepatocytes, ordered by culture time and cell type. In hepatocyte-like cells obtained after hAM-MSC differentiation, a decreased expression of the alpha-fetoprotein, a marker of fetal hepatocytes, was also observed [111]

Culture time & surface	Differentiating medium	Gene up- regulation	Increased protein expression	Cell activity	Ref.
human amniotic mesenchymal stromal cells (hAM-MSC)					
3 weeks plastic	DMEM/LG, 15% FBS, 20 ng/ml HGF, 10^-7^ M dex, 10 ng/ml OSM, ITS	Not given	AFP, CK18	Not given	[129]
collagen	1st step: basal medium, 2% FBS, 10 ng/ml bFGF, 20 ng/ml HGF, 0.61 g/L nicotinamide	-	-	-	[111]
	2nd step: basal medium, 2% FBS, 1 mM dex, 20 ng/ml OSM, 50 mg/ml ITS+ premix	ALB, CYP3A4	ALB, HGF	Glycogen storage, cellular uptake of indocyanine green	
collagen type-I	α-MEM, 10% FBS, 10 ng/mL hFGF-2, 20 ng/mL hHGF, 0.1 mmol/L dex, 10 ng/mL OSM	A1AT, AFP, CK18, HNF-4α	A1AT, ALB, CK18,	Glycogen storage	[112]
human amniotic fluid mesenchymal stem cells (hAF-MSC)					
3 weeks plastic	1st step: IMDM, 10 ng/ml bFGF, 20 ng/ml HGF, 0.1% DMSO	-	-	-	[130]
	2nd step: IMDM, 1 M dex, 20ng/ml OSM, 50 mg/ml ITS+ premix	ALB	Not given	LDL uptake	
4 weeks collagen type-I	1st step: 60% DMEM, 40% MCDB-201, 2% FBS, 1.623 mM glutamine, 0.03 mM nicotinamide, 1 mg/ml linoleic-acid, 0.1 mM L-ascorbic acid, 0.25 mM sodium pyruvate	-	-	-	[131]
	2nd step: basal medium, 10 ng/ml FGF-4	-	-	-	
	3rd step: basal medium, 20 ng/ml HGF 4th step: basal medium, 20 ng/ml HGF, 20 mg/l dex, ITS, 1 mM trichostatin A	AFP, ALB, C/EBPa, CK18, CYP1A1, HNF1-α	AFP, ALB, C/EBPa, CK18, CYP1A1, HNF1-α	Glycogen storage, urea synthesis	
human chorionic mesenchymal stem cells (hCMSC)					
3 weeks plastic	α-MEM, 12% FBS, 2 mM L-glutamine, 10 ng/ml FGF-4, 20 ng/ml HGF, 10^-7^ M dex, 10 ng/ml OSM, ITS, 10 mM N-acetyl-L-cysteine	A1AT, AFP, ALB	A1AT, AFP, ALB	Not given	[132]
collagen	1st step: basal medium, 2% FBS, 10 ng/ml bFGF, 20 ng/ml HGF, 0.61 g/L nicotinamide	-	-	-	[111]
	2nd step: basal medium, 2% FBS, 1 mM dex, 20 ng/ml OSM, 50 mg/ml ITS+ premix	ALB, CYP3A4	ALB, HGF, SCF	Glycogen storage, cellular uptake of indocyanine green

**Table 5:** Protocols for *in vitro* differentiation of mesenchymal stem cells isolated from human umbilical cord (hUC-MSC/hWJ-MSC) towards hepatocytes, ordered by culture time

Culture time & surface	Differentiating medium	Gene up- regulation	Increased protein expression	Cell activity	Ref.
7 days plastic	liver homogenate supernatant - (150 mg of liver with 1 mL of DMEM/F12)	Not given	AFP, CK18, TPH	CYP3A activity, albumin and urea synthesis	[133]
18 days collagen type-IV	1st step: low-glucose DMEM, 40% MCDB 201 medium, 2% FBS, 10 ng/ml bFGF, 20 ng/ml HGF	-	-	-	[134]
	2nd step: low-glucose DMEM, 40% MCDB 201 medium, 2% FBS, 1 μM dex, 20 ng/ml OSM, ITS+ Premix	A1AT, ALB, CYP3A4, HNF1-α	Not given	Indocyanine green uptake	
3 weeks collagen	1st step: basal medium, 2% FBS, 10 ng/ml bFGF, 20 ng/ml HGF, 0.61 g/L nicotinamide	-	-	-	[111]
	2nd step: basal medium, 2% FBS, 1 mM dex, 20 ng/ml OSM, 50 mg/ml ITS+ premix	ALB, CYP3A4	ALB, HGF	Glycogen storage, cellular uptake of indocyanine green	
plastic	IDMEM, 1% FBS, 10 ng/mL FGF-4, 40 ng/mL HGF	AFP, ALB, CK18	AFP, ALB, CK8	LDL uptake, glycogen synthesis	[135]
22 days plastic	1st step: ADMEM, 2% FBS, 20 ng/ml hHGF	-	-	-	[136]
	2nd step: ADMEM 2% FBS, 10 nmol/l dex, 10 ng/ml OSM, 1% ITS mix	ALB, HNF-4α	ALB, HNF-4α	Glycogen storage, urea synthesis, LDL uptake	
4 weeks plastic	1st step: 5 mM valproic acid	-	-	-	[137]
	2nd step: 2% FBS, 10 ng/mL FGF-4, 25 ng/mL HGF	-	-	-	
	3rd step: 2% FBS, 25 ng/mL HGF, 40 mg/mL dex, 20 ng/mL OSM, ITS premix	A1AT, A1AT, AFP, ALB, CK18, CYP1A1, CYP3A4, G6P, HNF-4α, TAT	ALB, CYP3A4	Glycogen storage, albumin and urea synthesis	
	1st step: IMDM, 10 ng/ml bFGF, 20 ng/ml HGF, 0.61 g/ml nicotinamide	-	-	-	[100]
	2nd step: 1 μmol/l dex, 20 ng/ml OSM, 50 mg/ml ITS	A1AT, AFP, ALB, CYP3A4, G6P, TAT	AFP, ALB, CYP3A4	Albumin secretion, increased blood urea nitrogen	

### Characteristics of Human Epithelial and Mesenchymal Afterbirth Cells

Human amniotic membrane is a source of both: epithelial (hAEC; human amniotic epithelial cells), and mesenchymal cells (hAM-MSC; human amniotic mesenchymal stromal cells). Population of epithelial cells can be obtained as a result of trypsinization of the amniotic membrane [103–106]. A part of hAEC has surface markers of pluripotency, such as SSEA-3, SSEA-4, TRA-1-60, TRA-1-81, and is characterized by the expression of the *OCT4*, *KLF4*, *REX1* genes [107, 108]. hAEC originate from the epiblast, which gives rise to three germ layers. This means, that isolated amnion epithelial cells can potentially give rise to tissues derived from all germ layers. The observed features of pluripotency make amnion epithelial cells similar to hESC. Furthermore, for ethical reasons and because of their biological properties, hAEC are an interesting alternative to embryonic cells in regenerative medicine. In contrast to hESC (or induced pluripotent stem cells - iPSC), they have low expression of MHC-I (major histocompatibility complex-I) antigens, and they lack MHC-II (major histocompatibility complex-II) antigens, what eliminates the possibility of inducing the recipient's immune system response. hAEC have low telomerase expression, and therefore do not exhibit tumorigenicity when used *in vivo* [12].

Amniotic mesenchymal cells, show much lower expression of markers for pluripotency than hAEC, and have higher expression of multipotency markers: CD44, CD49e, CD90, and CD105 [109]. Expression of multipotency markers can be observed also in other mesenchymal cells isolated from the afterbirth tissues. Their ability to differentiate into other cells is greater at the early stages of embryonic development and decreases over time. In general, afterbirth’s MSC potential to differentiate is higher than suggested by the presence of multipotency or pluripotency markers [110, 111]. *In vitro* studies have proved effective differentiation, particularly of hAM-MSC and hUC-MSC, towards adipocytic, chondrocytic, osteocytic, myogenic, angiogenic, cardiomyogenic, hepatocytic, pancreatocytic and neurocytic cell lines [111, 112].

#### Differentiation of Amniotic Epithelial Cells

Differentiation of hAEC towards functional hepatocytes can be performed by several methods, but the effectiveness of differentiation is difficult to compare between the research centers. Each of the leading research centers determines the degree of cell differentiation in a different way (Table 3). Although, the cells with features of fully mature hepatocytes have not been obtained yet, it has been indicated which one of the many signaling pathways is involved in this process [85].

Summing up publications describing the differentiation of amnion epithelial cells towards hepatocytes, the following conclusions can be made:

- So far, attempts to differentiate epithelial amnion cells have resulted in obtaining cells with features similar to fetal hepatocytes. It was proven based on experimental data of the CYP7A1/CYP3A4 expression ratio [114], and bigger secretion of alpha-fetoprotein over albumin [14, 114].
- Expression of genes characteristic for hepatocyte-like cells obtained in differentiation process is higher than in native hAEC, but definitely lower than in the positive control - HepG2 hepatic cell line [117].
- Culture on plates coated with extracellular matrix promotes hAEC differentiation into hepatocytes [115].

The presence of pluripotency markers, and the expression of genes responsible for differentiation and proliferation make amnion cells similar to induced pluripotent stem cells [118]. This similarity suggests the possibility of differentiating amnion cells with *in vitro* methods effective for iPSC [119–121]. Adopting effective differentiation protocols seems to be a promising methodological approach for hAEC differentiation [14, 122]. The first stage of this process is iPSC differentiation towards definitive endoderm cells, characterized by increased expression of *FOXA2* and *SOX17* genes [122, 123]. The following stages are: obtaining hepatoblasts, and their transformation into mature hepatocytes [124]. The cells obtained in this three-step protocol were described as functionally mature hepatocytes, however, different criteria for assessing the obtained cells have been used. Nevertheless, the obtained cells possess secretion, storage, metabolic abilities, and are able to repopulate a damaged liver in animal models [125]. Some preclinical trials have confirmed their usefulness in the treatment of acute liver failure [126] and congenital metabolic disorders associated with impaired protein synthesis [127]. On the other hand, there are concerns about the use of hepatic-like cells (HLC) obtained from iPSC in humans. It is uncertain, if the changes induced in the cell genome would not cause further spontaneous mutations leading to cancer. However, such concerns are not raised by the use of amnion epithelial cells [11] and, as it can be assumed, HLC obtained from them. Preclinical studies on mice [14, 113] using HLC obtained from hAEC have shown, that their use in the treatment of liver failure in humans does not require the use of immunosuppressive treatment [13].

#### Differentiation of Afterbirth Mesenchymal Cells

Afterbirth mesenchymal cells are an interesting alternative to hAEC for the purpose of hepatocyte differentiation (Tables 4 and 5). A certain limitation of their use on a larger scale is a small number of mesenchymal amniotic cells [109], or mesenchymal umbilical cord cells [128] isolated from one placenta as compared to hAEC [104].

Summing up the studies on differentiation of MSC isolated from afterbirth tissues towards hepatocytes, the following conclusions can be made:

- Media used in the differentiation of hAEC and MSC towards hepatocytes are similar in composition in terms of the growth factors used and their concentrations (Tables 3, 4 and 5). It is yet unknown whether hAEC or MSC have greater differentiation potential towards hepatocytes and this aspect requires further studies.
- Differentiated MSC display hepatocyte-like features *in vitro*, including urea and albumin synthesis, and CYP3A4 activity proven by proper diazepam and midazolam metabolism, which suggests functionality corresponding to mature hepatocytes [133].
- The differentiation process may be more efficient in afterbirth-derived MSC than in MSC of other origin [111].
- Sequential differentiation without prolonged exposure of MSC to HGF should be more effective than one step differentiation protocols with high concentrations of HGF. It is because prolonged exposure inhibits MSC proliferation and promotes changes in their cytoskeleton [138].
- 3D culture conditions are more effective in the differentiation process of MSC, than standard 2D culture [139].

## Preclinical Studies on Using Human Afterbirth Cells and Their Derivatives in Liver Failure Treatment

The potential outcomes of effective cell therapy performed with human stem cells isolated from the afterbirth tissues and their derivatives obtained during its *ex vivo* differentiation are: their documented potential to reduce inflammation, stimulate cell division, inhibit apoptosis, prevent fibrosis, and undergo further differentiation [105, 140]. A new scientific direction in the area of liver failure therapy are studies on exosomes secreted by stem cells into culture media. Exosomes are molecules other than growth factors, cytokines or hormones. They contain many microRNA, mRNA and protein molecules [141]. *In vivo* studies demonstrated the effectiveness of exosomes secreted by damaged hepatocytes [141], MSC [142] and hAEC [143] in suppression of toxic liver damage and its hepatoprotective activity.

### Preclinical Studies Conducted with hAEC

hAEC were used in the treatment of experimental liver failure in several animal models with native amniotic epithelial cells [144–147], hAEC culture media containing their secretory products [143], and epithelial cells partially differentiated towards human hepatocytes [113]. Each of these approaches has its advantages and limitations, nevertheless, they resulted in improved liver functioning (Table 6). Positive effects of experimental studies and the confirmed safety of amniotic epithelial cells in preclinical studies *in vivo,* formed the basis for conducting a case-control study in humans in which liver failure was treated with hAEC [11].

**Table 6:** Animal models of the use of hAEC, hAEC-derived hepatocytes and exosomes (EVs) in the treatment of liver failure

Host	Number of cells or EVs per one individual	Experimental model	Results	Ref.
Native hAEC				
Rat	3 × 10^6^	Acute injury – surgical BDL	Decreased: fibronectin deposition, areas occupied by myofibroblasts, number of cells positive for S100A4, TGFβ pathway activation, αvβ6 integrin expression.	[145]
Mouse	5 × 10^6^	Acute injury – CCl_4_	Decreased: levels of total bilirubin, ALAT, ASPAT, ALP; areas of liver necrosis. Increased: survival rate of mouse after lethal dose.	[113]
	4 x 10^6^	Chronic injury - NAFLD diet	Decreased: liver fibrosis, activation of HSC, activation of TGFβ pathway, amount and activity of liver parenchymal macrophages. General anti-fibrotic effect.	[144]
	0.5 x 10^6^	MPS1-knockout (Hurler syndrome)	Decreased: urinary GAG concentration, Increased: liver α-L-iduronidase enzyme activity, General therapeutic efficacy of hAEC for MPS1 Hurler syndrome.	[146]
hAEC-derived hepatocytes				
Mouse	5 × 10^6^	Acute injury - CCl_4_	Decreased: levels of total bilirubin, ALAT, ASPAT, ALP; areas of liver necrosis. Increased: survival rate of mice after the lethal dose.	[113]
hAEC exosomes				
Mouse	24 × 10^6^ hAEC-EVs	Chronic injury - CCl_4_	Decreased: TGFβ1 and α-SMA expression, collagen production, HSC activation, activation of Kupffer cells and a change in their phenotype. General immonomodulatory and anti-fibrotic effect.	[143]

Summing up the results of experiments performed in animal models *in vivo*, it can be stated that hAEC and their derivatives show the following effects in the acute and chronic liver injury:

- hAEC reduce TGFβ synthesis. This leads to inhibition of myofibroblasts derived from HSC, thereby reducing ECM production [145].
- Substances secreted by hAEC into their niche act anti-fibrotically in a paracrine manner [148, 149]. hAECs stimulate the transformation of macrophages into the M2 phenotype, which in contrast to M1, display immunomodulatory, anti-inflammatory and anti-fibrotic activity [143].
- The therapeutic effect of hAEC may be significantly dependent on the number of cells given to the recipient, what should constitute a subject of further studies (Table 6).

Positive effects of hAEC and their derivatives, such as improved liver function, reduction of fibrosis and inflammation, raise the question about the necessity of stem cells differentiation towards hepatocytes, if the use of native amniotic cells gives promising results. Unfortunately, it is not possible to give a clear answer to this question, due to a small number of scientific reports dealing with such cells in *in vivo* therapy. Lin et al. administered hAEC, partially differentiated towards hepatocytes to mice with chronic liver injury and achieved a greater increase in serum albumin levels in animals, and generally a stronger liver function improvement compared to those, that received native amniotic epithelial cells [14]. This indicates, that pre-differentiation towards a specific cell line may result in more effective regeneration and improvement of liver function. A similar phenomenon has been observed in case of preliminary *in vitro* differentiation of mesenchymal stem cells derived from adipose tissue [150], hESC [151] and iPSC [152]. Some researchers remark the aspect of the involvement of mature target organ cells in the *in vivo* differentiation of native hAEC [115, 153].

### Preclinical Studies Conducted with Mesenchymal Stem Cells

Mesenchymal cell populations isolated from the afterbirth tissues are well characterized in terms of their positive pleiotropic effect in the treatment of liver failure in animals (Tables 7 and 8) and in humans [154, 155].

**Table 7:** Animal models of the use of mesenchymal stem cells isolated from human umbilical cord (hUC-MSC/(hWJ-MSC), their exosomes (EVs) and umbilical cord blood (hUCB-MSC) in the treatment of liver failure

Host	Number of cells or exosomes per one individual	Experimental model	Results	Ref.
human umbilical cord mesenchymal stem cells (hUC-MSC/(hWJ-MSC)				
Rat	5 × 10^6^	Dimethylnitrosamine-induced liver fibrosis	Decreased: ALAT and ASPAT plasma levels, and fibrosis, cholestasis, collagen deposition in the liver; Increased: mobilization of Kupffer cells, transition from M1 to M2 phenotype, and IL-10 and IL-4 plasma levels.	[156]
	5 × 10^6^	Chronic injury – CCl_4_	Decreased: expression of α-SMA, TIMP-1, collagen type I and III, and ALAT, ASPAT and plasma bilirubin levels; hepatocyte swelling, necrosis, steatosis and centers of regeneration; Increased: expression of vimentin, E-cadherin, α-catenin and MMP-13, and ALB concentration.	[157]
	3 x 10^6^ hUC-MSC ------------- 2.85-3mg hUC-MSC-EVs	Ischaemia/reperfusion (I/R) injury	Decreased: ALAT, ASPAT, ALP, IL-1β, IL-6, and TNFα in plasma, hepatocyte necrosis, number of hepatic infiltrating neutrophils, expression of caspase 3 and mitochondrial reactive oxygen species levels, serum serum IL-1β, IL-6, and TNFα levels (all in both groups - hUC-MSC-treated and hUC-MSC-EVs-treated) Decreased: mRNA levels for IL-1β, IL-6, TNFα, CC motif ligand 12, IFN-γ and TLR4 (in hUC-MSC-EVs-treated group)	[142]
	2.2-2.5 x 10^6^	Acute injury - D-GalN (1000 mg/kg b.w.) and LPS (10μg/kg b.w.)	Decreased: ALAT, ASPAT and bilirubin in plasma, hepatocyte necrosis and inflammation, number of apoptotic hepatocytes; Increased: number of proliferating hepatocytes.	[158]
	1 x 10^6^	Acute injury – CCl_4_	Decreased: ALAT, ASPAT and bilirubin levels in plasma, inflammation, cell degeneration and necrosis.	[133]
Mouse	5 × 10^6^	Acute injury - acetaminofen i.p. 500mg/kg b.w.	Decreased: ALAT, ASPAT, ALP, GGTP and bilirubin in plasma, interstitial inflammation.	[159]
	3-5 x 10^6^	Acute injury - CCl_4_	Decreased ALAT, ASPAT and bilirubin levels.	[160]
	2 x 10^6^	Chronic injury – CCl_4_	Decreased: ALAT and ASPAT plasma levels, COL1, COL3, TGF-β1 mRNA expression, inflammation and swelling of liver cells, damage to mitochondria and parenchyma; Increased: TGFα mRNA expression.	[161]
	1 × 10^6^	Ischaemia/reperfusion (I/R) injury	Decreased: ALAT and ASPAT in plasma, severity of damage.	[162]
	5 x 10^5^	Fulminant injury – D-GalN	Decreased: number of necrotic cells and inflammatory response cells, reduced liver damage, extended survival time of animals.	[163]
	2.5 x 10^5^	Acute injury – CCl_4_	ALAT, MCP-1 and IP-10 serum levels were not significantly changed indicating lack of therapeutic effect of stem cells.	[164]
human umbilical cord blood mesenchymal stem cells (hUCB-MSC)				
Mouse	1 x 10^6^	Chronic injury – CCl_4_	Decreased: bilirubin level, expression of ITGB1 and COL1A1, number of SMA(+) cells and parenchymal fibrosis; Increased: albumin synthesis.	[165]

**Table 8:** Animal models of the use of mesenchymal stem cells isolated from human placenta in the treatment of liver failure

Host	Number of cells or exosomes per one individual	Experimental model	Results	Ref.
human amniotic mesenchymal stromal stem cells (hAM-MSC)				
Rat	1 x 10^6^	Sclerosing cholangitis, ANIT-induced	Decreased: biliary hyperplasia, fewer necrotic changes, expression of MMP-9, α-SMA, TGF-β, type I collagen, MMP-2 and TIMP-1, Glisson score.	[166]
		Chronic injury – CCl_4_ induced	Decreased: expression of COL1, α-SMA, CD68 and TIMP-1; Increased: expression of MMP-9 and HGF. General anti-fibrotic effect.	[167]
Mouse	intrahepatic administration, number not given	Acute injury - CCl_4_ induced	Decreased: ALAT, ASPAT, TNF and IFN-γ plasma levels, histological markers of liver damage; Increased: IL-10 plasma concentration.	[168]
human chorionic plate mesenchymal stem cells (hCP-MSC)				
Rat	2 × 10^6^	Chronic injury - CCl_4_ induced	Decreased: expression of Shh, Smo, Gli2 and Gli3 (HH signaling pathway), amount of hepatic hydroxyproline, number of collagen fibers and liver progenitor cells; Increased: expression of Ihh protein and miRNA-125b.	[169]
			Decreased: IL-6 and IL-6 receptor methylation; Increased: expression of cyclins E and A, albumin, and IL-6; methylation of SOCS3 and STAT3.	[170]
			Decreased: lymphocyte infiltration, expression of PARP, caspase 3/7, proteins of the pathway associated with autophagy (PI3K class III, Beclin1, ATG7, ATG5-12, and LC3 II), and phosphorylated m-TOR kinase; Increased: HIF-1α expression and its translocation to the nucleus, protein expression of Bcl-2, Bax, factors associated with proliferation (Jak1, PI3K p110a, phosphorylated ERK1/2, and Smad2/3), cyclin E and A, PTTG1, IL-6, gp130, ABCG1 and ABCG2, and proliferation index (Ki67).	[171]

Attempts have been made to evaluate *in vivo* effects of hepatocyte-like cells derived from afterbirth’s mesenchymal stem cells (Table 9), however this issue requires further studies, ideally comparing the effectiveness of HLC derived from mesenchymal stem cells and native MSC, in the treatment of liver failure.

**Table 9:** Animal models of the use of hepatocyte-like cells derived from human umbilical cord and umbilical cord blood in the treatment of liver failure

Host	Number of HLCsper one individual	Experimental model	Results	Ref.
human umbilical cord-derived hepatocyte-like cells				
Rat	1 x 10^6^	Acute injury - CCl_4_	Decreased: bilirubin, ALAT and ASPAT serum levels, reduced liver damage; Increased: serum albumin level. survival rate of rats after lethal dose.	[172]
Mouse			Decreased: ALAT and ASPAT serum levels, reduced liver damage; Increased: serum albumin level.	[173]
human umbilical cord blood-derived hepatocyte-like cells				
Mouse	1 x 10^6^	Chronic injury – CCl_4_	Decreased: ALAT and ASPAT plasma levels, fibrotic index. Reduced liver damage.	[174]

Summarizing the results of preclinical experiments *in vivo*, the following conclusions can be made regarding the effect of mesenchymal afterbirth cells in acute and chronic liver failure therapy:

- MSC reduce the inflammatory process associated with liver damage by reducing the expression and secretion of inflammatory cytokines. Moreover, they increase the expression and thereby secretion of anti-inflammatory cytokines [156, 175].
- Using MSC in therapy results in limited liver fibrosis [110, 166, 167].
- MSC affect the phenotype of M1 macrophages and promote their change into M2 [175].
- Mesenchymal cells isolated from the postnatal tissues reduce the cytokine storm phenomenon during acute and chronic liver damage, thus reducing the cell apoptosis and necrotic areas [133, 142, 157, 158, 176, 177].
- HLC obtained from MSC differentiation under 3D culture conditions are more effective in lowering transaminase levels after *in vivo* use, than those differentiated under 2D conditions [174].
- Hepatocytes derived from MSC improve liver function and increase serum albumin levels more efficiently than native MSC [173].
- *In vivo* use of MSC partially differentiated into hepatocytes appears to be safer than use of native MSC due to the fact that the use of native MSC may result in their differentiation into fibroblasts, which gather in fibrous septa, thus acting profibrotically [172, 178].

## Clinical Studies on Epithelial and Mesenchymal Afterbirth Cells in the Treatment of Liver Failure

Based on preclinical attempts to treat liver failure using stem cells isolated from the afterbirth tissues, it can be concluded that these cells support the natural regenerative mechanisms. The use of afterbirth cells is intended to improve liver function, rather than to rebuild its structure. Native cells improve liver function by reducing inflammation and fibrosis, and as a consequence the remaining organ parenchyma has a chance to regenerate itself and regain metabolic function including the synthesis of proteins. However, the use of native cells resulted in lesser improvement compared to partially differentiated cells. Positive results and the lack of side effects of the use of native hAEC in preclinical studies of liver failure therapy resulted in their introduction into human studies. First stages of clinical trials were carried out on patients in the terminal stage of chronic liver failure. They aimed at the assessment of safety of native hAEC, but not epithelial cells differentiated into hepatocytes, in therapy of liver diseases in humans, but their results have not yet been published [11, 13].

Over the last decade, the interest in mesenchymal cells has increased significantly, and the total number of registered clinical trials using native hAM-MSC, hAF-MSC, hWJ-MSC, hCP-MSC, hCV-MSC and hUC-MSC in the treatment of various diseases, exceeded 100 [75]. According to the U.S. National Library of Medicine database, 20 clinical trials using hUC-MSC are registered for the treatment of liver diseases, including 15 in cirrhosis (as of 12/03/2020). In the study of Xu et al., hUC-MSC (1 x 10^5^ cells/kg b.w. twice or four times, depending on the group) were administered to patients with acute hepatic failure overlapping chronic failure caused by HBV infection. The decrease in ALAT, ASPAT and bilirubin in plasma, as well as in MELD score, was observed [154]. In the pilot study of Shi et al., patients after liver transplantation with acute transplant rejection received hUC-MSC (1 x 10^6^ cells/kg b.w.) together with standard immunosuppression. Patients after hUC-MSC infusion had lower ALAT plasma levels, and increased levels of the TGFβ1 and PGE2, in comparison to the control group. Moreover, improved liver morphology and a higher number of regulatory T lymphocytes in peripheral blood, were observed. No side effects of stem cell infusions were identified [179]. Zhang et al. examined the effects of hUC-MSC in patients with decompensated cirrhosis in the course of chronic hepatitis B. Patients received 0.5 x 10^6^ cells/kg b.w. and were followed up for two years after administration. Increased levels of plasma albumin, prothrombin and cholinesterase, together with reduced total bilirubin level were observed in patients in the study group. The authors concluded about possible improvements in liver function and thrombin functionality in these patients. The decrease in the MELD Na score was faster in the patients in the study group, and a significant reduction in ascites was observed. The concentration of liver fibrosis markers (serum laminin, hyaluronic acid, PIIINP and type IV collagen) decreased and HGF level (fibrosis inhibiting factor) increased compared to the control group [155]. Unfortunately, trials using afterbirth MSC other than hUC-MSC or MSC partially differentiated into hepatic cells are not registered for the treatment of liver diseases [75].

## Summary

Previous *in vitro* and *in vivo* studies indicate that cells isolated from the afterbirth tissues may play an important role in the supportive therapy for acute and chronic liver failure. Their advantages include multilineage potential for differentiation, no need for immunosuppression after administration, and no tumor formation in recipient organisms [147, 180]. The heterogeneous population of these cells comprises the cells of different stages of differentiation, namely: pluripotent, multipotent, progenitor, and mature cells, characterized by the expression of specific transcription factors and surface markers [181]. The expression of epithelial, or mesenchymal markers, as well as lineage-associated markers of early differentiation, varies among hAEC and afterbirth MSC reflecting their potential to proliferation, self-renewal, and differentiation towards the cells representing three germ lineages. Both hAEC and MSC express embryonic stem cell and pluripotency markers, however the level of marker expression remains variable. Furthermore, hAEC express epithelial cell markers such as cytokeratins, E-cadherin, and EpCAM. In turn, mesenchymal cell markers are more specific to afterbirth MSC but they have also been reported on the surface of hAEC [182, 183]. A very small amount or none of the studied hAEC and afterbirth MSC express hematopoietic markers. However, both these populations express various endodermal markers, as GATA-4, HNF-3β, AFP, ALB (in hAEC), GLUT-2, CK18, A1AT, and HNF-4α. After the culture of amnion cells with the hepatic differentiation protocol, they expressed high mRNA levels of both fetal and adult hepatocyte markers: AFP, CYP7A1, ALB, and A1AT [85]. It should be noted, that there are discrepancies in the data presented by authors in the literature concerning the pattern of markers expressed by epithelial and mesenchymal afterbirth cells [109, 181].

Native human afterbirth cells share some phenotypic features comparable with human hepatic multipotent stem cells (hHpSC) and bipotent hepatoblasts: EpCAM, CK19, CD133 [17, 27, 35, 181], adult multipotent stem cells expressing mesenchymal markers: CD44, CD73, CD90, CD105, CK19, SSEA-4, NANOG, OCT-4 [15, 52, 66, 67, 181, 53–60] and adult bipotent hepatic progenitor cells (HPC): DLK-1 [78, 184]. EpCAM expression, characteristic of hepatoblasts, but not of mesenchymal liver cells, has been confirmed in afterbirth cells only in few studies [181, 183–185].

**Fig. 1:**
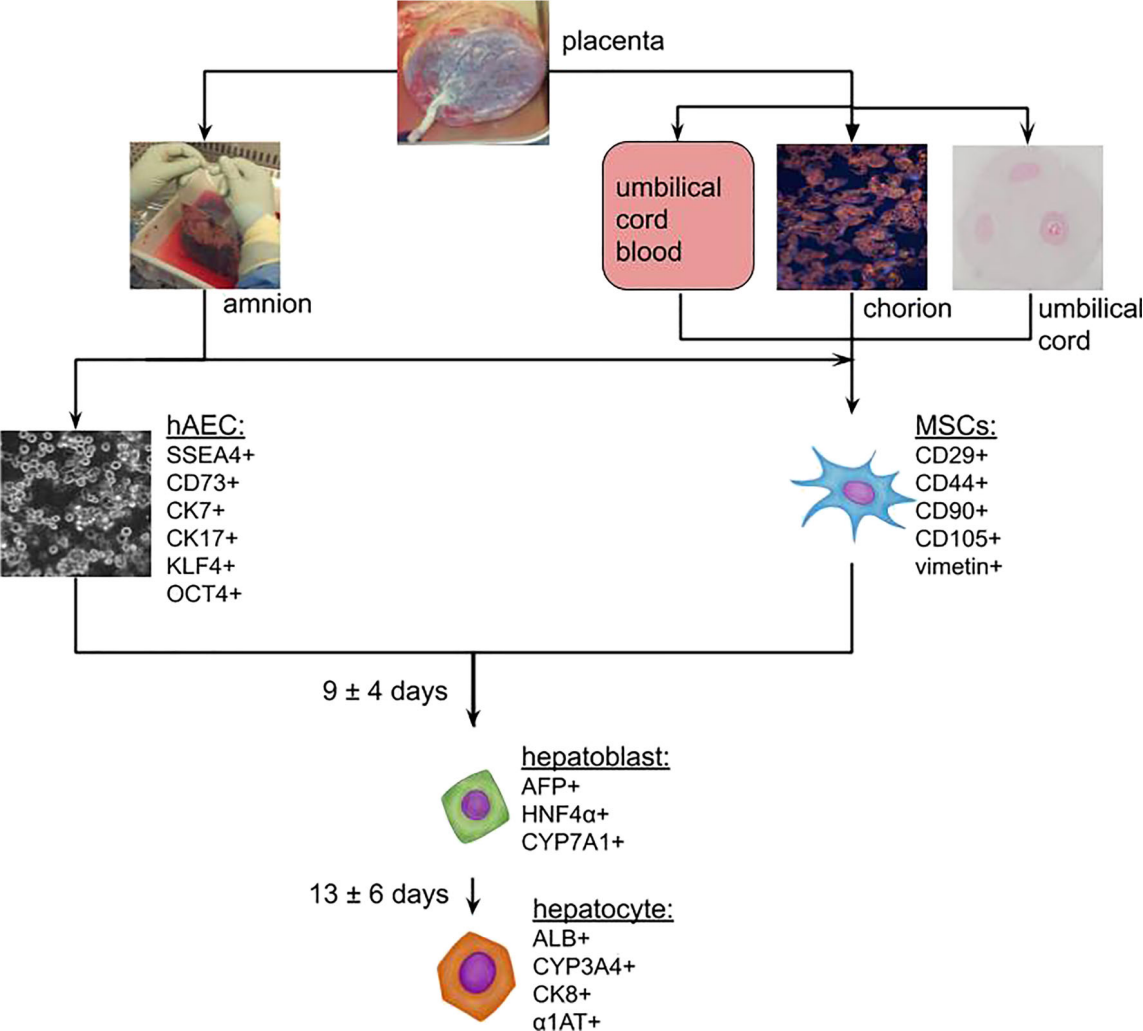
Stages of *in vitro* differentiation of cells isolated from afterbirth tissues towards hepatocyte-like cells. Attempts to pre-differentiate afterbirth stem cells prior to transplantation have resulted in obtaining mainly hepatocytes displaying fetal properties. The duration time of each stage and specific cell markers are given according to the literature data cited in the text and tables. Photographs were made in the Department of Cytophysiology, Medical University of Silesia

Experimental data indicate that stem cell pre-differentiation towards a specific cell line may result in more effective regeneration and improvement of liver function [14]. So far, attempts to differentiate afterbirth cells toward hepatocytes resulted in obtaining cells with features of hepatoblasts, characterized by elevated expression of fetal hepatocyte-specific genes (Fig. 1). It is yet unknown whether hAEC or afterbirth MSC have greater hepatocytic differentiation potential. It cannot be excluded that the differentiation process may be more efficient in afterbirth-derived MSC than in MSC of other origins. Sequential differentiation without prolonged exposure of MSC to HGF should be more effective than one step differentiation protocols with high concentrations of HGF. Culturing the afterbirth cells on plates coated with extracellular matrix or 3D culture conditions promote differentiation into hepatocytes. A similar phenomenon of enhanced liver regeneration by pre-differentiated stem cells [14] has been observed during preliminary *in vitro* differentiation of adipose tissue-derived mesenchymal stem cells, hESC, and iPSC [126]. Perhaps the use of a three-step differentiation protocol for the differentiation of the fetal MSC and/or hAEC will result in fully functional hepatocytes, as has been described earlier in iPSC cultures [122].

Preclinical trials using the native afterbirth MSC and hAEC resulted in improved liver function in animals, and the cells given became similar to hepatocytes over time, thus, to some extent, restored its structure [180]. Afterbirth cells reduced the inflammatory process associated with liver damage by inhibiting the production of inflammatory cytokines and secretion of anti-inflammatory cytokines, thus reducing the cell apoptosis and necrotic areas, and inhibited liver fibrosis [145, 156, 175]. Positive effects observed in animals led to clinical trials in humans. In the case of native hUC-MSC, beneficial effects on liver function were observed, whereas results of the use of native hAEC are yet unknown [11, 13].

The use of pre-differentiated human liver cells in preclinical studies resulted in noticeably better improvement in liver function, compared to the native cells [14]. However, the protocols for *in vitro* obtaining hepatocytes from afterbirth stem cells are not optimized and require further improvements and subsequent evaluation of the usefulness of these cells in *in vivo* liver regeneration models. To date, no clinical trial using hepatocyte-like cells obtained by differentiation of afterbirth cells, based on effective, safe, and GMP-compliant protocol cells, has been registered.

## References

[1] Adam R, Karam V, Cailliez VO, Grady JG, Mirza D, Cherqui D, Klempnauer J, Salizzoni M, Pratschke J, Jamieson N, Hidalgo E, Paul A, Lopez Andujar R, Lerut J, Fisher L, Boudjema K, Fondevila C, Soubrane O, Bachellier P, Pinna AD, Berlakovich G, Bennet W, Pinzani M, Schemmer P, Zieniewicz K, Jimenez Romero C, De Simone P, Ericzon B, Schneeberger S, Wigmore SJ, Fabregat Prous J, Colledan M, Porte RJ, Yilmaz S, Azoulay D, Pirenne J, Line P, Trunecka P, Navarro F, Valdivieso Lopez A, De Carlis L, Rufian Pena S, Kochs E, Duvoux C (2018). 2018 Annual Report of the European Liver Transplant Registry (ELTR) – 50-year evolution of liver transplantation. Transplant International.

[2] Kubota T, Hata K, Sozu T, Ueda Y, Hirao H, Okamura Y, Tamaki I, Yoshikawa J, Kusakabe J, Tanaka H, Kageyama S, Anazawa T, Yoshizawa A, Yagi S, Yamashiki N, Okajima H, Kaido T, Uemoto S (2018). Impact of donor age on recipient survival in adult-to-adult living-donor liver transplantation. Annals of Surgery.

[3] Emre S, Umman V (2011). Split liver transplantation: an overview. Transplantation Proceedings.

[4] Gilgenkrantz H, Collin de l’Hortet, A. (2018). Understanding liver regeneration: from mechanisms to regenerative medicine. American Journal of Pathology.

[5] Iansante V, Mitry RR, Filippi C, Fitzpatrick E, Dhawan A (2018). Human hepatocyte transplantation for liver disease: current status and future perspectives. Pediatric Research.

[6] Hu J, Yu X, Wang Z, Wang F, Wang L, Gao H, Chen Y, Zhao W, Jia Z, Yan S, Wang Y (2013). Long term effects of the implantation of Wharton’s jelly-derived mesenchymal stem cells from the umbilical cord for newly-onset type 1 diabetes mellitus. Endocrine Journal.

[7] Wang D, Feng X, Lu L, Konkel JE, Zhang H, Chen Z, Li X, Gao X, Lu L, Shi S, Chen W, Sun L (2014). A CD8 T cell/indoleamine 2,3-dioxygenase axis is required for mesenchymal stem cell suppression of human systemic lupus erythematosus. Arthritis and Rheumatology.

[8] Liang J, Sun L (2015). Mesenchymal stem cells transplantation for systemic lupus erythematosus. International Journal of Rheumatic Diseases.

[9] Wu X, Xia Y, Zhou O, Song Y, Zhang X, Tian D, Li Q, Shu C, Liu E, Yuan X, He L, Liu C, Li J, Liang X, Yang K, Fu Z, Zou L, Bao L, Dai J (2020). Allogeneic human umbilical cord-derived mesenchymal stem cells for severe bronchopulmonary dysplasia in children: study protocol for a randomized controlled trial (MSC-BPD trial). Trials.

[10] Phan TG, Ma H, Lim R, Sobey CG, Wallace EM (2018). Phase 1 trial of amnion cell therapy for ischemic stroke. Frontiers in Neurology.

[11] Lim R, Hodge A, Moore G, Wallace EM, Sievert W (2017). A pilot study evaluating the safety of intravenously administered human amnion epithelial cells for the treatment of hepatic fibrosis. Frontiers in Pharmacology.

[12] Strom SC, Gramignoli R (2016). Human amnion epithelial cells expressing HLA-G as novel cell-based treatment for liver disease. Human Immunology.

[13] Gramignoli R, Morandi F, Horenstein A, Strunz B, Srinivasan RC, Malavasi F, Strom SC (2019). Cellular mechanism in support of allogenic human amnion epithelial cell transplantation without immunosuppression. Cytotherapy.

[14] Lin JS, Zhou L, Sagayaraj A, Jumat NHB, Choolani M, Chan JKY, Biswas A, Wong PC, Lim SG, Dan YY (2015). Hepatic differentiation of human amniotic epithelial cells and in vivo therapeutic effect on animal model of cirrhosis. Journal of Gastroenterology and Hepatology (Australia).

[15] Kholodenko IV, Kurbatov LK, Kholodenko RV, Manukyan GV, Yarygin KN (2019). Mesenchymal stem cells in the adult human liver: hype or hope?. Cells.

[16] Tanimizu N, Mitaka T (2014). Re-evaluation of liver stem / progenitor cells. Organogenesis.

[17] Schmelzer E (2019). Hepatic progenitors of the fetal liver: interactions with hematopoietic stem cells. Differentiation.

[18] Katoonizadeh, A. (2017). *Liver regeneration. Liver pathophysiology: Therapies and antioxidants*. Elsevier Inc. 10.1016/B978-0-12-804274-8.00007-2.

[19] Ko S, Russell JO, Molina LM, Monga SP (2020). Liver progenitors and adult cell plasticity in hepatic injury and repair: knowns and unknowns. Annual Review of Pathology: Mechanisms of Disease.

[20] Yang L, Li LC, Lamaoqiezhong, Wang X, Wang WH, Wang YC, Xu CR (2019). The contributions of mesoderm-derived cells in liver development. Seminars in Cell and Developmental Biology.

[21] Trefts E, Gannon M, Wasserman DH (2017). The liver. Current Biology.

[22] Gordillo M, Evans T, Gouon-Evans V (2015). Orchestrating liver development. Development (Cambridge).

[23] Shin D, Pal S, Monga S (2013). Cellular and molecular basis of liver development anteroposterior endoderm patterning. Comprehensive Physiology.

[24] Chaudhari P, Tian L, Deshmukh A, Jang YY (2016). Expression kinetics of hepatic progenitor markers in cellular models of human liver development recapitulating hepatocyte and biliary cell fate commitment. Experimental Biology and Medicine.

[25] Yang L, Wang WH, Qiu WL, Guo Z, Bi E, Xu CR (2017). A single-cell transcriptomic analysis reveals precise pathways and regulatory mechanisms underlying hepatoblast differentiation. Hepatology.

[26] Gruppuso PA, Sanders JA (2016). Regulation of liver development: implications for liver biology across the lifespan. Journal of Molecular Endocrinology.

[27] Schmelzer E, Zhang L, Bruce A, Wauthier E, Ludlow J, Yao HL, Moss N, Melhem A, McClelland R, Turner W, Kulik M, Sherwood S, Tallheden T, Cheng N, Furth ME, Reid LM (2007). Human hepatic stem cells from fetal and postnatal donors. Journal of Experimental Medicine.

[28] Carpino G, Cardinale V, Onori P, Franchitto A, Berloco PB, Rossi M, Wang Y, Semeraro R, Anceschi M, Brunelli R, Alvaro D, Reid LM, Gaudio E (2012). Biliary tree stem/progenitor cells in glands of extrahepatic and intraheptic bile ducts: an anatomical in situ study yielding evidence of maturational lineages. Journal of Anatomy.

[29] Cardinale V, Wang Y, Carpino G, Cui CB, Gatto M, Rossi M, Berloco PM, Cantafora A, Wauthier E, Furth ME, Inverardi L, Dominguez-Bendala J, Ricordi C, Gerber D, Gaudio E, Alvaro D, Reid LM (2011). Multipotent stem/progenitor cells in human biliary tree give rise to hepatocytes, cholangiocytes, and pancreatic islets. Hepatology.

[30] Keller L, Werner S, Pantel K (2019). Biology and clinical relevance of EpCAM. Cell Stress.

[31] Semeraro R, Cardinale V, Carpino G, Gentile R, Napoli C, Venere R, Gatto M, Brunelli R, Gaudio E, Alvaro D (2013). The fetal liver as cell source for the regenerative medicine of liver and pancreas. Annals of Translational Medicine.

[32] Goldman O, Cohen I, Gouon-Evans V (2016). Functional blood progenitor markers in developing human liver progenitors. Stem Cell Reports.

[33] Tanaka M, Okabe M, Suzuki K, Kamiya Y, Tsukahara Y, Saito S, Miyajima A (2009). Mouse hepatoblasts at distinct developmental stages are characterized by expression of EpCAM and DLK1: drastic change of EpCAM expression during liver development. Mechanisms of Development.

[34] Lombard CA, Prigent J, Sokal EM (2013). human liver progenitor cells for liver repair. Cell Medicine.

[35] Schmelzer E, Wauthier E, Reid LM (2006). The phenotypes of pluripotent human hepatic progenitors. Stem Cells.

[36] Segal, J. M., Kent, D., Wesche, D. J., Ng, S. S., Serra, M., Oulès, B., Kar, G., Emerton, G., Blackford, S. J. I., Darmanis, S., Miquel, R., Luong, T. V., Yamamoto, R., Bonham, A., Jassem, W., Heaton, N., Vigilante, A., King, A., Sancho, R., Teichmann, S., Quake, S. R., Nakauchi, H., & Rashid, S. T. (2019). Single cell analysis of human foetal liver captures the transcriptional profile of hepatobiliary hybrid progenitors. *Nature Communications, 10*(1). 10.1038/s41467-019-11266-x. 10.1038/s41467-019-11266-x PMC6659636 31350390

[37] Fomin, M. E., Beyer, A. I., & Muench, M. O. (2017). Human fetal liver cultures support multiple cell lineages that can engraft immunodeficient mice. *Open Biology, 7*(12). 10.1098/rsob.170108. 10.1098/rsob.170108 PMC5746544 29237808

[38] Turner R, Lozoya O, Wang Y, Cardinale V, Gaudio E, Alpini G, Mendel G, Wauthier E, Barbier C, Alvaro D, Reid LM (2011). Human hepatic stem cell and maturational liver lineage biology. Hepatology.

[39] Dianat N, Dubois-Pot-Schneider H, Steichen C, Desterke C, Leclerc P, Raveux A, Combettes A, Weber A, Corlu A, Dubart-Kupperschmitt A (2014). Generation of functional cholangiocyte-like cells from human pluripotent stem cells and HepaRG cells. Hepatology.

[40] Tabibian JH, Trussoni CE, O’Hara SP, Splinter PL, Heimbach JK, LaRusso NF (2014). Characterization of cultured cholangiocytes isolated from livers of patients with primary sclerosing cholangitis. Laboratory Investigation.

[41] Pietrosi G, Vizzini G, Gerlach J, Chinnici C, Luca A, Amico G, D’Amato M, Conaldi PG, Petri SL, Spada M, Tuzzolino F, Alio L, Schmelzer E, Gridelli B (2015). Phases I–II matched case-control study of human fetal liver cell transplantation for treatment of chronic liver disease. Cell Transplantation.

[42] Valizadeh A, Majidinia M, Samadi-Kafil H, Yousefi M, Yousefi B (2019). The roles of signaling pathways in liver repair and regeneration. Journal of Cellular Physiology.

[43] Michalopoulos GK (2017). Hepatostat: liver regeneration and normal liver tissue maintenance. Hepatology.

[44] Tao, Y., Wang, M., Chen, E., & Tang, H. (2017). Liver regeneration: analysis of the main relevant signaling molecules. *Mediators of Inflammation*, *2017*. 10.1155/2017/4256352 10.1155/2017/4256352 PMC5602614 28947857

[45] Michalopoulos GK (2010). Liver regeneration after partial hepatectomy: critical analysis of mechanistic dilemmas. American Journal of Pathology.

[46] Cordero-Espinoza L, Huch M (2018). The balancing act of the liver: tissue regeneration versus fibrosis. Journal of Clinical Investigation.

[47] Forbes SJ, Newsome PN (2016). Liver regeneration-mechanisms and models to clinical application. Nature Reviews Gastroenterology and Hepatology.

[48] Tanaka W, Yamanaka N, Oriyama T, Katoh T, Kuroda N, Okamoto E (1997). Multivariate analysis of liver regenerative capacity after hepatectomy in humans. Journal of Hepato-Biliary-Pancreatic Surgery.

[49] Ibis C, Asenov Y, Akin M, Azamat IF, Sivrikoz N, Gurtekin B (2017). Factors affecting liver regeneration in living donors after hepatectomy. Medical Science Monitor.

[50] Avila MA, Moschetta A (2015). The FXR-FGF19 gut-liver axis as a novel “hepatostat”. Gastroenterology.

[51] Michalopoulos GK (2014). Advances in liver regeneration. Expert Review of Gastroenterology and Hepatology.

[52] Herrera MB, Bruno S, Buttiglieri S, Tetta C, Gatti S, Deregibus MC, Bussolati B, Camussi G (2006). Isolation and characterization of a stem cell population from adult human liver. Stem Cells.

[53] Navarro-Tableros V, Herrera Sanchez MB, Figliolini F, Romagnoli R, Tetta C, Camussi G (2015). Recellularization of rat liver scaffolds by human liver stem cells. Tissue Engineering - Part A.

[54] Bruno, S., Grange, C., Tapparo, M., Pasquino, C., Romagnoli, R., Dametto, E., Amoroso, A., Tetta, C., & Camussi, G. (2016). Human liver stem cells suppress T-cell proliferation, NK activity, and dendritic cell differentiation. *Stem Cells International, 2016*. 10.1155/2016/8468549. 10.1155/2016/8468549 PMC4834412 27127520

[55] Herrera MB, Fonsato V, Bruno S, Grange C, Gilbo N, Romagnoli R, Tetta C, Camussi G (2013). Human liver stem cells improve liver injury in a model of fulminant liver failure. Hepatology.

[56] Navarro-Tableros V, Gai C, Gomez Y, Giunti S, Pasquino C, Deregibus MC, Tapparo M, Pitino A, Tetta C, Brizzi MF, Ricordi C, Camussi G (2019). Islet-like structures generated in vitro from adult human liver stem cells revert hyperglycemia in diabetic SCID mice. Stem Cell Reviews and Reports.

[57] Bruno, S., Herrera Sanchez, M. B., Pasquino, C., Tapparo, M., Cedrino, M., Tetta, C., & Camussi, G. (2019). Human liver-derived stem cells improve fibrosis and inflammation associated with nonalcoholic steatohepatitis. *Stem Cells International, 2019*. 10.1155/2019/6351091. 10.1155/2019/6351091 PMC6589210 31281379

[58] Lopatina T, Grange C, Fonsato V, Tapparo M, Brossa A, Fallo S, Pitino A, Herrera-Sanchez MB, Kholia S, Camussi G, Bussolati B (2019). Extracellular vesicles from human liver stem cells inhibit tumor angiogenesis. International Journal of Cancer.

[59] Spada M, Porta F, Righi D, Gazzera C, Tandoi F, Ferrero I, Fagioli F, Herrera Sanchez MB, Calvo PG, Biamino E, Bruno S, Gunetti M, Contursi C, Lauritano C, Conio A, Amoroso A, Salizzoni M, Silengo L, Camussi G, Romagnoli R (2020). Intrahepatic administration of human liver stem cells in infants with inherited neonatal-onset hyperammonemia: a phase I study. Stem Cell Reviews and Reports.

[60] Famulari ES, Navarro-Tableros V, Herrera Sanchez MB, Bortolussi G, Gai M, Conti L, Silengo L, Tolosano E, Tetta C, Muro AF, Camussi G, Fagoonee S, Altruda F (2020). Human liver stem cells express UGT1A1 and improve phenotype of immunocompromised Crigler Najjar syndrome type I mice. Scientific Reports.

[61] Lee JH, Park HJ, Kim YA, Lee DH, Noh JK, Kwon CHD, Jung SM, Lee SK (2012). Differentiation and major histocompatibility complex antigen expression in human liver-derived stem cells. Transplantation Proceedings.

[62] Lee JH, Park HJ, Kim YA, Lee DH, Noh JK, Kwon CHD, Jung SM, Lee SK (2012). The phenotypic characteristic of liver-derived stem cells from adult human deceased donor liver. Transplantation Proceedings.

[63] Lee JH, Park HJ, Jang IK, Kim HE, Lee DH, Park JK, Lee SK, Yoon HH (2014). In vitro differentiation of human liver-derived stem cells with mesenchymal characteristics into immature hepatocyte-like cells. Transplantation Proceedings.

[64] Najimi M, Khuu DN, Lysy PA, Jazouli N, Abarca J, Sempoux C, Sokal EM (2007). Adult-derived human liver mesenchymal-like cells as a potential progenitor reservoir of hepatocytes?. Cell Transplantation.

[65] Pan Q, Fouraschen SMG, Kaya FSFA, Verstegen MM, Pescatori M, Stubbs AP, Van Ijcken W, Van Der Sloot A, Smits R, Kwekkeboom J, Metselaar HJ, Kazemier G, De Jonge J, Tilanus HW, Wagemaker G, Janssen HLA, Van Der Laan LJW (2011). Mobilization of hepatic mesenchymal stem cells from human liver grafts. Liver Transplantation.

[66] Kholodenko IV, Kholodenko RV, Manukyan GV, Burunova VV, Yarygin KN (2016). Mesenchymal-epithelial transition in culture of stromal progenitor cells isolated from the liver of a patient with alcoholic cirrhosis. Bulletin of Experimental Biology and Medicine.

[67] Kholodenko IV, Kholodenko RV, Manukyan GV, Yarygin KN (2017). Hepatic differentiation of adult and fetal liver stromal cells in vitro. Biochemistry (Moscow) Supplement Series B: Biomedical Chemistry.

[68] Bruno S, Pasquino C, Herrera Sanchez MB, Tapparo M, Figliolini F, Grange C, Chiabotto G, Cedrino M, Deregibus MC, Tetta C, Camussi G (2020). HLSC-derived extracellular vesicles attenuate liver fibrosis and inflammation in a murine model of non-alcoholic steatohepatitis. Molecular Therapy.

[69] Sanchez MBH, Bruno S, Grange C, Tapparo M, Cantaluppi V, Tetta C, Camussi G (2014). Human liver stem cells and derived extracellular vesicles improve recovery in a murine model of acute kidney injury. Stem Cell Research and Therapy.

[70] Kholia, S., Herrera Sanchez, M. B., Cedrino, M., Papadimitriou, E., Tapparo, M., Deregibus, M. C., Brizzi, M. F., Tetta, C., & Camussi, G. (2018). Human liver stem cell-derived extracellular vesicles prevent aristolochic acid-induced kidney fibrosis. *Frontiers in Immunology, 9*(JUL). 10.3389/fimmu.2018.01639. 10.3389/fimmu.2018.01639 PMC6060249 30072992

[71] Rigo F, De Stefano N, Navarro-Tableros V, David E, Rizza G, Catalano G, Gilbo N, Maione F, Gonella F, Roggio D, Martini S, Patrono D, Salizzoni M, Camussi G, Romagnoli R (2018). Extracellular vesicles from human liver stem cells reduce injury in an ex vivo normothermic hypoxic rat liver perfusion model. Transplantation.

[72] Grange C, Tritta S, Tapparo M, Cedrino M, Tetta C, Camussi G, Brizzi MF (2019). Stem cell-derived extracellular vesicles inhibit and revert fibrosis progression in a mouse model of diabetic nephropathy. Scientific Reports.

[73] Brossa A, Fonsato V, Grange C, Tritta S, Tapparo M, Calvetti R, Cedrino M, Fallo S, Gontero P, Camussi G, Bussolati B (2020). Extracellular vesicles from human liver stem cells inhibit renal cancer stem cell-derived tumor growth in vitro and in vivo. International Journal of Cancer.

[74] Fonsato V, De Lena M, Tritta S, Brossa A, Calvetti R, Tetta C, Camussi G, Bussolati B (2018). Human liver stem cell-derived extracellular vesicles enhance cancer stem cell sensitivity to tyrosine kinase inhibitors through Akt/mTOR/PTEN combined modulation. Oncotarget.

[75] U.S. National Library of Medicine. (2020). Retrieved August 12, 2020, from www.clinicaltrials.gov

[76] Abdellatif, H. (2018). Oval cells: potential role in liver regeneration. *Biomedical Journal of Scientific & Technical Research, 2*(1). 10.26717/bjstr.2018.02.000665.

[77] Fausto N, Campbell JS (2003). The role of hepatocytes and oval cells in liver regeneration and repopulation. Mechanisms of Development.

[78] Shin S, Kaestner KH (2014). The origin, biology, and therapeutic potential of facultative adult hepatic progenitor cells. Current Topics in Developmental Biology.

[79] Czekaj P (2004). Molecular and cellular mechanisms of chemically induced hepatocarcinogenesis. Polish Journal of Environmental Studies.

[80] Tanaka M, Miyajima A (2016). Liver regeneration and fibrosis after inflammation. Inflammation and Regeneration.

[81] Fausto N (2004). Liver regeneration and repair: hepatocytes, progenitor cells, and stem cells. Hepatology.

[82] Spee B, Carpino G, Schotanus BA, Katoonizadeh A, Vander Borght S, Gaudio E, Roskams T (2010). Characterisation of the liver progenitor cell niche in liver diseases: Potential involvement of Wnt and Notch signalling. Gut.

[83] Tsuchiya A, Yu W (2019). Liver stem cells: plasticity of the liver epithelium. World Journal of Gastroenterology.

[84] Ji T, Li G, Chen J, Zhao J, Li X, Lin H, Cai X, Cang Y (2016). Distinct role of interleukin-6 and tumor necrosis factor receptor-1 in oval cell- mediated liver regeneration and inflammationassociated hepatocarcinogenesis. Oncotarget.

[85] Maymó JL, Riedel R, Pérez-Pérez A, Magatti M, Maskin B, Dueñas JL, Parolini O, Sánchez-Margalet V, Varone CL (2018). Proliferation and survival of human amniotic epithelial cells during their hepatic differentiation. PLoS ONE.

[86] Oh S-H, Hatch HM, Petersen BE (2002). Hepatic oval ‘stem’ cell in liver regeneration. Seminars in Cell & Developmental Biology.

[87] Carpino G, Morini S, Carotti S, Gaudio E (2020). Hepatic progenitor cells and biliary tree stem cells. Liver diseases.

[88] López-Navarrete G, Ramos-Martínez E, Suárez-Álvarez K, Aguirre-García J, Ledezma-Soto Y, León-Cabrera S, Gudiño-Zayas M, Guzmán C, Gutiérrez-Reyes G, Hernández-Ruíz J, Camacho-Arroyo I, Robles-Díaz G, Kershenobich D, Terrazas LI, Escobedo G (2011). Th2-associated alternative Kupffer Cell activation promotes liver fibrosis without inducing local inflammation. International Journal of Biological Sciences.

[89] Li J, Xin J, Zhang L, Wu J, Jiang L, Zhou Q, Li J, Guo J, Hongcui C, Li L (2013). Human hepatic progenitor cells express hematopoietic cell markers CD45 and CD109. International Journal of Medical Sciences.

[90] Grompe M (2014). Liver stem cells, where art thou?. Cell Stem Cell.

[91] Ji H, Lu Y, Shi Y (2017). Seeds in the liver. Acta Histochemica.

[92] Tarlow BD, Pelz CN, Wakefield WEL, Elizabeth M, Finegold MJ, Grompe M (2014). Bipotential adult liver progenitors are derived from chronically injured mature hepatocytes. Cell Stem Cell.

[93] Overi, D., Carpino, G., Cardinale, V., Franchitto, A., Safarikia, S., Onori, P., Alvaro, D., & Gaudio, E. (2018). Contribution of resident stem cells to liver and biliary tree regeneration in human diseases. *International Journal of Molecular Sciences, 19*(10). 10.3390/ijms19102917. 10.3390/ijms19102917 PMC6213374 30257529

[94] Fabregat, I., Moreno-Càceres, J., Sánchez, A., Dooley, S., Dewidar, B., Giannelli, G., & ten Dijke, P. (2016). TGF-β signalling and liver disease. *FEBS Journal*, 2219–2232. 10.1111/febs.13665. 10.1111/febs.13665 26807763

[95] Chen YG, Lui HM, Lin SL, Lee JM, Ying SY (2002). Regulation of cell proliferation, apoptosis, and carcinogenesis by activin. Experimental Biology and Medicine.

[96] Chen J, Tschudy-Seney B, Ma X, Zern MA, Liu P, Duan Y (2018). Salvianolic acid B enhances hepatic differentiation of human embryonic stem cells through upregulation of WNT pathway and inhibition of Notch pathway. Stem Cells and Development.

[97] Li Q, Hutchins AP, Chen Y, Li S, Shan Y, Liao B, Zheng D, Shi X, Li Y, Chan W, Pan G, Wei S, Shu X, Pei D (2017). A sequential EMT-MET mechanism drives the differentiation of human embryonic stem cells towards hepatocytes. Nature Communications.

[98] Han YJ, Kang YH, Shivakumar SB, Bharti D, Son YB, Choi YH, Park WU, Byun JH, Rho GJ, Park BW (2017). Stem cells from cryopreserved human dental pulp tissues sequentially differentiate into definitive endoderm and hepatocyte-like cells in vitro. International Journal of Medical Sciences.

[99] Kim HJ, Cho YA, Lee YM, Lee SY, Bae WJ, Kim EC (2016). PIN1 suppresses the hepatic differentiation of pulp stem cells via Wnt3a. Journal of Dental Research.

[100] Yu YB, Song Y, Chen Y, Zhang F, Qi FZ (2018). Differentiation of umbilical cord mesenchymal stem cells into hepatocytes in comparison with bone marrow mesenchymal stem cells. Molecular Medicine Reports.

[101] Wang B, Li W, Dean D, Mishra MK, Wekesa KS (2018). Enhanced hepatogenic differentiation of bone marrow derived mesenchymal stem cells on liver ECM hydrogel. Journal of Biomedical Materials Research - Part A.

[102] Fiore EJ, Mazzolini G, Aquino JB (2015). Mesenchymal stem/stromal cells in liver fibrosis: recent findings, old/new caveats and future perspectives. Stem Cell Reviews and Reports.

[103] Miki, T., Marongiu, F., Dorko, K., Ellis, E. C. S., & Strom, S. C. (2010). Isolation of amniotic epithelial stem cells. *Current Protocols in Stem Cell Biology*, 1–10. 10.1002/9780470151808.sc01e03s12. 10.1002/9780470151808.sc01e03s12 20049689

[104] Gramignoli R, Srinivasan RC, Kannisto K, Strom SC (2016). Isolation of human amnion epithelial cells according to current good manufacturing procedures. Current Protocols in Stem Cell Biology.

[105] Kolanko E, Kopaczka K, Koryciak-Komarska H, Czech E, Szmytkowska P, Gramignoli R, Czekaj P (2019). Increased immunomodulatory capacity of human amniotic cells after activation by pro-inflammatory chemokines. European Journal of Pharmacology.

[106] Szmytkowska, P., Koryciak-Komarska, H., Limanówka, Ł., Król, M., Plewka, D., Kopaczka, K., & Czekaj, P. (2019). Comparison on efficiency of various enzymatic methods for the isolation of cells from human amnion. In *Advances in biomedical research. From microbiology to cancer* (pp. 99–109). Lublin: Tygiel, Warszawa: Warszawski Uniwersytet Medyczny.

[107] García-Castro IL, García-López G, Ávila-González D, Flores-Herrera H, Molina-Hernández A, Portillo W, Ramón-Gallegos E, Díaz NF (2015). Markers of pluripotency in human amniotic epithelial cells and their differentiation to progenitor of cortical neurons. PLoS ONE.

[108] Parolini O, Alviano F, Bagnara GP, Bilic G, Bühring H-J, Evangelista M, Hennerbichler S, Liu B, Magatti M, Mao N, Miki T, Marongiu F, Nakajima H, Nikaido T, Portmann-Lanz CB, Sankar V, Soncini M, Stadler G, Surbek D, Takahashi TA, Redl H, Sakuragawa N, Wolbank S, Zeisberger S, Zisch A, Strom SC (2008). Concise review: isolation and characterization of cells from human term placenta: outcome of the first international workshop on placenta derived stem cells. Stem Cells.

[109] Bilic G, Zeisberger SM, Mallik AS, Zimmermann R, Zisch AH (2008). Comparative characterization of cultured human term amnion epithelial and mesenchymal stromal cells for application in cell therapy. Cell Transplantation.

[110] Kim EY, Lee KB, Kim MK (2014). The potential of mesenchymal stem cells derived from amniotic membrane and amniotic fluid for neuronal regenerative therapy. BMB Reports.

[111] Lee HJ, Jung J, Cho KJ, Lee CK, Hwang SG, Kim GJ (2012). Comparison of in vitro hepatogenic differentiation potential between various placenta-derived stem cells and other adult stem cells as an alternative source of functional hepatocytes. Differentiation.

[112] Tamagawa T, Oi S, Ishiwata I, Ishikawa H, Nakamura Y (2007). Differentiation of mesenchymal cells derived from human amniotic membranes into hepatocyte-like cells in vitro. Human Cell.

[113] Liu QW, Liu QY, Li JY, Wei L, Ren KK, Zhang XC, Ding T, Xiao L, Zhang WJ, Wu HY, Xin HB (2018). Therapeutic efficiency of human amniotic epithelial stem cell-derived functional hepatocyte-like cells in mice with acute hepatic failure. Stem Cell Research and Therapy.

[114] Miki, T., Marongiu, F., Ellis, E. C. S., Dorko, K., Mitamura, K., Ranade, A., Gramignoli, R., Davila, J., & Strom, S. C. (2009). Production of hepatocyte-like cells from human amnion. In *Hepatocyte transplantation* (Vol. 481, pp. 155–168). 10.1007/978-1-59745-201-4_13 10.1007/978-1-59745-201-4_13 19096803

[115] Marongiu F, Gramignoli R, Dorko K, Miki T, Ranade AR, Serra MP, Doratiotto S, Sini M, Sharma S, Mitamura K, Sellaro TL, Tahan V, Skvorak KJ, Ellis ECS, Badylak SF, Davila JC, Hines R, Laconi E, Strom SC (2011). Hepatic differentiation of amniotic epithelial cells. Hepatology.

[116] Vaghjiani V, Vaithilingam V, Saraswati I, Sali A, Murthi P, Kalionis B, Tuch BE, Manuelpillai U (2014). Hepatocyte-like cells derived from human amniotic epithelial cells can be encapsulated without loss of viability or function in vitro. Stem Cells and Development.

[117] Tee JY, Vaghjiani V, Han Liu Y, Murthi P, Chan J, Manuelpillai U (2013). Immunogenicity and immunomodulatory properties of hepatocyte-like cells derived from human amniotic epithelial cells. Current Stem Cell Research & Therapy.

[118] Miki T, Marongiu F, Ellis E, Strom C, S. (2007). Isolation of amniotic epithelial stem cells. Current Protocols in Stem Cell Biology.

[119] Starokozhko V, Hemmingsen M, Larsen L, Mohanty S, Merema M, Pimentel RC, Wolff A, Emneus J, Aspergen A, Groothius G, Dufva M (2018). Differentiation of human-induced pluripotent stem cell under flow conditions to mature hepatocytes for liver tissue engineering. Journal of Tissue Engineering and Regenerative Medicine.

[120] Xia Y, Carpentier A, Cheng X, Block PD, Zhao Y, Zhang Z, Protzer U, Liang TJ (2017). Human stem cell-derived hepatocytes as a model for hepatitis B virus infection, spreading and virus-host interactions. Journal of Hepatology.

[121] Song W, Lu YC, Frankel AS, An D, Schwartz RE, Ma M (2015). Engraftment of human induced pluripotent stem cell-derived hepatocytes in immunocompetent mice via 3D co-aggregation and encapsulation. Scientific Reports.

[122] Carpentier A, Nimgaonkar I, Chu V, Xia Y, Hu Z, Liang TJ (2016). Hepatic differentiation of human pluripotent stem cells in miniaturized format suitable for high-throughput screen. Stem Cell Research.

[123] Tasnim, F., Phan, D., Toh, Y. C., & Yu, H. (2015). Cost-effective differentiation of hepatocyte-like cells from human pluripotent stem cells using small molecules. *Biomaterials* (Vol. 70). Elsevier Ltd. 10.1016/j.biomaterials.2015.08.002 10.1016/j.biomaterials.2015.08.002 26310107

[124] Palakkan AA, Nanda J, Ross JA (2017). Pluripotent stem cells to hepatocytes, the journey so far. Biomedical Reports.

[125] Hannoun Z, Steichen C, Dianat N, Weber A, Dubart-Kupperschmitt A (2016). The potential of induced pluripotent stem cell derived hepatocytes. Journal of Hepatology.

[126] Takayama K, Akita N, Mimura N, Akahira R, Taniguchi Y, Ikeda M, Sakurai F, Ohara O, Morio T, Sekiguchi K, Mizuguchi H (2017). Generation of safe and therapeutically effective human induced pluripotent stem cell-derived hepatocyte-like cells for regenerative medicine. Hepatology Communications.

[127] Okamoto R, Takayama K, Akita N, Nagamoto Y, Hosokawa D, Iizuka S, Sakurai F, Suemizu H, Ohashi K, Mizuguchi H (2018). Human iPS cell–based liver-like tissue engineering at extrahepatic sites in mice as a new cell therapy for hemophilia B. Cell Transplantation.

[128] Han YF, Tao R, Sun TJ, Chai JK, Xu G, Liu J (2013). Optimization of human umbilical cord mesenchymal stem cell isolation and culture methods. Cytotechnology.

[129] Resca E, Zavatti M, Maraldi T, Bertoni L, Beretti F, Guida M, La Sala GB, Guillot PV, David AL, Sebire NJ, De Pol A, De Coppi P (2015). Enrichment in c-Kit improved differentiation potential of amniotic membrane progenitor/stem cells. Placenta.

[130] Roubelakis MG, Pappa KI, Bitsika V, Zagoura D, Vlahou A, Papadaki HA, Antsaklis A, Anagnou NP (2007). Molecular and proteomic characterization of human mesenchymal stem cells derived from amniotic fluid: comparison to bone marrow mesenchymal stem cells. Stem Cells and Development.

[131] Zheng YB, Gao ZL, Xie C, Zhu HP, Peng L, Chen JH, Chong YT (2008). Characterization and hepatogenic differentiation of mesenchymal stem cells from human amniotic fluid and human bone marrow: a comparative study. Cell Biology International.

[132] Nazarov I, Lee JW, Soupene E, Etemad S, Knapik D, Green W, Bashkirova E, Fang X, Matthay MA, Kuypers FA, Serikov VB (2012). Multipotent stromal stem cells from human placenta demonstrate high therapeutic potential. STEM CELLS Translational Medicine.

[133] Xue G, Han X, Ma X, Wu H, Qin Y, Liu J, Hu Y, Hong Y, Hou Y (2016). Effect of microenvironment on differentiation of human umbilical cord mesenchymal stem cells into hepatocytes in vitro and in vivo. BioMed Research International.

[134] Kim MJ, Shin KS, Jeon JH, Lee DR, Shim SH, Kim JK, Cha DH, Yoon TK, Kim GJ (2011). Human chorionic-plate-derived mesenchymal stem cells and Wharton’s jelly-derived mesenchymal stem cells: a comparative analysis of their potential as placenta-derived stem cells. Cell and Tissue Research.

[135] Zhang YN, Lie PC, Wei X (2009). Differentiation of mesenchymal stromal cells derived from umbilical cord Wharton’s jelly into hepatocyte-like cells. Cytotherapy.

[136] Bharti D, Shivakumar SB, Park JK, Ullah I, Subbarao RB, Park JS, Lee SL, Park BW, Rho GJ (2018). Comparative analysis of human Wharton’s jelly mesenchymal stem cells derived from different parts of the same umbilical cord. Cell and Tissue Research.

[137] Raut A, Khanna A (2016). Enhanced expression of hepatocyte-specific microRNAs in valproic acid mediated hepatic trans-differentiation of human umbilical cord derived mesenchymal stem cells. Experimental Cell Research.

[138] Forte G, Minieri M, Cossa P, Antenucci D, Sala M, Gnocchi V, Fiaccavento R, Carotenuto F, De Vito P, Morena Baldini P, Prat M, Di Nardo P (2006). Hepatocyte growth factor effects on mesenchymal stem cells: proliferation, migration, and differentiation. Stem Cells.

[139] Afshari A, Shamdani S, Uzan G, Naserian S, Azarpira N (2020). Different approaches for transformation of mesenchymal stem cells into hepatocyte-like cells. Stem Cell Research and Therapy.

[140] Kopaczka K, Skowron K, Kolanko E, Czekaj P (2016). The relationship between amniotic epithelial cells and their microenvironment. Journal of Applied Biomedicine.

[141] Chen L, Chen R, Kemper S, Brigstock DR (2018). Pathways of production and delivery of hepatocyte exosomes. Journal of Cell Communication and Signaling.

[142] Yao J, Zheng J, Cai J, Zeng K, Zhou C, Zhang J, Li S, Li H, Chen L, He L, Chen H, Fu H, Zhang Q, Chen G, Yang Y, Zhang Y (2019). Extracellular vesicles derived from human umbilical cord mesenchymal stem cells alleviate rat hepatic ischemia-reperfusion injury by suppressing oxidative stress and neutrophil inflammatory response. FASEB Journal.

[143] Alhomrani M, Correia J, Zavou M, Leaw B, Kuk N, Xu R, Saad MI, Hodge A, Greening DW, Lim R, Sievert W (2017). The human amnion epithelial cell secretome decreases hepatic fibrosis in mice with chronic liver fibrosis. Frontiers in Pharmacology.

[144] Kuk N, Hodge A, Sun Y, Correia J, Alhomrani M, Samuel C, Moore G, Lim R, Sievert W (2019). Human amnion epithelial cells and their soluble factors reduce liver fibrosis in murine non-alcoholic steatohepatitis. Journal of Gastroenterology and Hepatology (Australia).

[145] Cargnoni A, Farigu S, Cotti Piccinelli E, Bonassi Signoroni P, Romele P, Vanosi G, Toschi I, Cesari V, Barros Sant’Anna L, Magatti M, Silini AR, Parolini O (2018). Effect of human amniotic epithelial cells on pro-fibrogenic resident hepatic cells in a rat model of liver fibrosis. Journal of Cellular and Molecular Medicine.

[146] Rodriguez NS, Yanuaria L, Parducho KMR, Garcia IM, Varghese BA, Grubbs BH, Miki T (2017). Liver-directed human amniotic epithelial cell transplantation improves systemic disease phenotype in hurler syndrome mouse model. Stem Cells Translational Medicine.

[147] Czekaj P, Król M, Limanówka Ł, Michalik M, Lorek K, Gramignoli R (2019). Assessment of animal experimental models of toxic liver injury in the context of their potential application as preclinical models for cell therapy. European Journal of Pharmacology.

[148] Zhao B, Liu JQ, Yang C, Zheng Z, Zhou Q, Guan H, Su LL, Hu DH (2016). Human amniotic epithelial cells attenuate TGF-β1-induced human dermal fibroblast transformation to myofibroblasts via TGF-β1/Smad3 pathway. Cytotherapy.

[149] Hodge A, Lourensz D, Vaghjiani V, Nguyen H, Tchongue J, Wang B, Murthi P, Sievert W, Manuelpillai U (2014). Soluble factors derived from human amniotic epithelial cells suppress collagen production in human hepatic stellate cells. Cytotherapy.

[150] Aurich H, Sgodda M, Kaltwaßer P, Vetter M, Weise A, Liehr T, Brulport M, Hengstler JG, Dollinger MM, Fleig WE, Christ B (2009). Hepatocyte differentiation of mesenchymal stem cells from human adipose tissue in vitro promotes hepatic integration in vivo. Gut.

[151] Li, Z., Wu, J., Wang, L., Han, W., Yu, J., Liu, X., Wang, Y., Zhang, Y., Feng, G., Li, W., Stacey, G. N., Gu, Q., Hu, B., Wang, L., Zhou, Q., & Hao, J. (2019). Generation of qualified clinical-grade functional hepatocytes from human embryonic stem cells in chemically defined conditions. *Cell Death and Disease, 10*(10). 10.1038/s41419-019-1967-5. 10.1038/s41419-019-1967-5 PMC6787193 31601782

[152] Balasiddaiah A, Moreno D, Guembe L, Prieto J, Aldabe R (2013). Hepatic differentiation of mouse iPS cells and analysis of liver engraftment potential of multistage iPS progeny. Journal of Physiology and Biochemistry.

[153] Maraldi T, Bertoni L, Riccio M, Zavatti M, Carnevale G, Resca E, Guida M, Beretti F, La Sala GB, De Pol A (2014). Human amniotic fluid stem cells: neural differentiation in vitro and in vivo. Cell and Tissue Research.

[154] Xu, W. Z., He, H. L., Pan, S. W., Chen, Y. L., Zhang, M. L., Zhu, S., Gao, Z. L., Peng, L., & Li, J. G. (2019). Combination treatments of plasma exchange and umbilical cord-derived mesenchymal stem cell transplantation for patients with hepatitis B virus-related acute-on-chronic liver failure: a clinical trial in China. *Stem Cells International, 2019*. 10.1155/2019/4130757. 10.1155/2019/4130757 PMC6378797 30863450

[155] Zhang Z, Lin H, Shi M, Xu R, Fu J, Lv J, Li Y, Yu S, Geng H, Jin L, Lau GK, Wang FS (2012). Human umbilical cord mesenchymal stem cells improve liver function and ascites in decompensated liver cirrhosis patients. Journal of Gastroenterology and Hepatology (Australia).

[156] Chai NL, Zhang XB, Chen SW, Fan KX, Linghu EQ (2016). Umbilical cord-derived mesenchymal stem cells alleviate liver fibrosis in rats. World Journal of Gastroenterology.

[157] Zhang GZ, Sun HC, Zheng LB, Guo JB, Zhang XL (2017). In vivo hepatic differentiation potential of human umbilical cord-derived mesenchymal stem cells: therapeutic effect on liver fibrosis/cirrhosis. World Journal of Gastroenterology.

[158] Zhang, Y., Li, Y., Li, W., Cai, J., Yue, M., Jiang, L., Xu, R., Zhang, L., Li, J., & Zhu, C. (2018). Therapeutic effect of human umbilical cord mesenchymal stem cells at various passages on acute liver failure in rats. *Stem Cells International, 2018*. 10.1155/2018/7159465. 10.1155/2018/7159465 PMC6261392 30538751

[159] Chetty, S. S., Praneetha, S., Govarthanan, K., Verma, R. S., & Vadivel Murugan, A. (2019). Noninvasive tracking and regenerative capabilities of transplanted human umbilical cord-derived mesenchymal stem cells labeled with I-III-IV Semiconducting Nanocrystals In Liver-Injured Living mice.*, ACS Applied Materials and Interfaces, 11*(9), 8763–8778. research-article. 10.1021/acsami.8b19953. 10.1021/acsami.8b19953 30741534

[160] Feng T, Zhang J, Zeng G, Zhou R, Tang X, Cui C, Li Y, Wang H, Li T, Zhu W, Yu Z (2015). Therapeutic potential of umbilical cord mesenchymal stem cells in mice with acute hepatic failure. International Journal of Artificial Organs.

[161] Cui H, Liu Z, Wang L, Bian Y, Li W, Zhou H, Chu X, Zhao Q (2017). Icariin-treated human umbilical cord mesenchymal stem cells decrease chronic liver injury in mice. Cytotechnology.

[162] Zheng J, Li H, He L, Huang Y, Cai J, Chen L, Zhou C, Fu H, Lu T, Zhang Y, Yao J, Yang Y (2019). Preconditioning of umbilical cord-derived mesenchymal stem cells by rapamycin increases cell migration and ameliorates liver ischaemia/reperfusion injury in mice via the CXCR4/CXCL12 axis. Cell Proliferation.

[163] Yang JF, Cao HC, Pan QL, Yu J, Li J, Li LJ (2015). Mesenchymal stem cells from the human umbilical cord ameliorate fulminant hepatic failure and increase survival in mice. Hepatobiliary and Pancreatic Diseases International.

[164] De Witte SFH, Merino AM, Franquesa M, Strini T, Van Zoggel JAA, Korevaar SS, Luk F, Gargesha M, O’Flynn L, Roy D, Elliman SJ, Newsome PN, Baan CC, Hoogduijn MJ (2017). Cytokine treatment optimises the immunotherapeutic effects of umbilical cord-derived MSC for treatment of inflammatory liver disease. Stem Cell Research and Therapy.

[165] Van Le T, Nguyen NH, Do HQ, Le HM, Truong NH (2017). Transplantation of umbilical cord blood-derived mesenchymal stem cells to treat liver cirrhosis in mice: a comparison of tail and portal vein injection. Progress in Stem Cell.

[166] Sugiura R, Ohnishi S, Ohara M, Ishikawa M, Miyamoto S, Onishi R, Yamamoto K, Kawakubo K, Kuwatani M, Sakamoto N (2018). Effects of human amnion-derived mesenchymal stem cells and conditioned medium in rats with sclerosing cholangitis. American Journal of Translational Research.

[167] Kubo K, Ohnishi S, Hosono H, Fukai M, Kameya A, Higashi R, Yamada T, Onishi R, Yamahara K, Takeda H, Sakamoto N (2015). Human amnion-derived mesenchymal stem cell transplantation ameliorates liver fibrosis in rats. Transplantation Direct.

[168] Zagoura D, Trohatou O, Makridakis M, Kollia A, Kokla N, Mokou M, Psaraki A, Eliopoulos AG, Vlahou A, Roubelakis MG (2019). Functional secretome analysis reveals Annexin-A1 as important paracrine factor derived from fetal mesenchymal stem cells in hepatic regeneration. EBioMedicine.

[169] Hyun J, Wang S, Kim J, Kim GJ, Jung Y (2015). MicroRNA125b-mediated Hedgehog signaling influences liver regeneration by chorionic plate-derived mesenchymal stem cells. Scientific Reports.

[170] Jung J, Moon JW, Choi JH, Lee YW, Park SH, Kim GJ (2015). Epigenetic alterations of IL-6/STAT3 signaling by placental stem cells promote hepatic regeneration in a rat model with CCl4-induced liver injury. International Journal of Stem Cells.

[171] Jung J, Choi JH, Lee Y, Park JW, Oh IH, Hwang SG, Kim KS, Kim GJ (2013). Human placenta-derived mesenchymal stem cells promote hepatic regeneration in CCl4-injured rat liver model via increased autophagic mechanism. Stem Cells.

[172] Kao S, Shyu J, Wang H, Hsiao C, Su C, Chen T, Weng Z (2016). Transplantation of hepatocyte-like cells derived from umbilical cord stromal mesenchymal stem cells to treat acute liver failure rat abstract. Journal of Biomedical Sciences.

[173] Cui L, Shi Y, Zhou X, Wang X, Wang J, Lan Y, Wang M, Zheng L, Li H, Wu Q, Zhang J, Fan D, Han Y (2013). A set of microRNAs mediate direct conversion of human umbilical cord lining-derived mesenchymal stem cells into hepatocytes. Cell Death and Disease.

[174] El Baz H, Demerdash Z, Kamel M, Hammam O, Samir Abdelhady D, Mahmoud S, Hassan S, Mahmoud F, Atta S, Riad NM, Gaafar T (2020). Induction of hepatic regeneration in an experimental model using hepatocyte-differentiated mesenchymal stem cells. Cellular Reprogramming.

[175] Eom YW, Shim KY, Baik SK (2015). Mesenchymal stem cell therapy for liver fibrosis. The Korean Journal of Internal Medicine.

[176] Guo G, Zhuang X, Xu Q, Wu Z, Zhu Y, Zhou Y, Li Y, Lu Y, Zhang B, Talbot P, Liao J, She J, Bu H, Shi Y (2019). Peripheral infusion of human umbilical cord mesenchymal stem cells rescues acute liver failure lethality in monkeys. Stem Cell Research and Therapy.

[177] Tsuchiya A, Takeuchi S, Watanabe T, Yoshida T, Nojiri S, Ogawa M, Terai S (2019). Mesenchymal stem cell therapies for liver cirrhosis: MSCs as “conducting cells” for improvement of liver fibrosis and regeneration. Inflammation and Regeneration.

[178] Valfrè Di Bonzo L, Ferrero I, Cravanzola C, Mareschi K, Rustichell D, Novo E, Sanavio F, Cannito S, Zamara E, Bertero M, Davit A, Francica S, Novelli F, Colombatto S, Fagioli F, Parola M (2008). Human mesenchymal stem cells as a two-edged sword in hepatic regenerative medicine: engraftment and hepatocyte differentiation versus profibrogenic potential. Gut.

[179] Shi M, Liu Z, Wang Y, Xu R, Sun Y, Zhang M, Yu X, Wang H, Meng L, Su H, Jin L, Wang FS (2017). A pilot study of mesenchymal stem cell therapy for acute liver allograft rejection. Stem Cells Translational Medicine.

[180] Gramignoli R (2016). Therapeutic use of human amnion-derived products: cell-based therapy for liver disease. Current Pathobiology Reports.

[181] Ghamari SH, Abbasi-Kangevari M, Tayebi T, Bahrami S, Niknejad H (2020). The bottlenecks in translating placenta-derived amniotic epithelial and mesenchymal stromal cells into the clinic: current discrepancies in marker reports. Frontiers in Bioengineering and Biotechnology.

[182] Koike C, Zhou K, Takeda Y, Fathy M, Okabe M, Yoshida T, Nakamura Y, Kato Y, Nikaido T (2014). Characterization of amniotic stem cells. Cellular Reprogramming.

[183] Topoluk N, Hawkins R, Tokish J, Mercuri J (2017). Amniotic mesenchymal stromal cells exhibit preferential osteogenic and chondrogenic differentiation and enhanced matrix production compared with adipose mesenchymal stromal cells. The American Journal of Sports Medicine.

[184] Srinivasan RC, Strom SC, Gramignoli R (2020). Effects of cryogenic storage on human amnion epithelial cells. Cells.

[185] Pratama, G., Vaghjiani, V., Tee, J. Y., Liu, Y. H., Chan, J., Tan, C., Murthi, P., Gargett, C., & Manuelpillai, U. (2011). Changes in culture expanded human amniotic epithelial cells: implications for potential therapeutic applications. *PLoS ONE, 6*(11). 10.1371/journal.pone.0026136. 10.1371/journal.pone.0026136 PMC3206797 22073147

